# Protective Strategies of *Haberlea rhodopensis* for Acquisition of Freezing Tolerance: Interaction between Dehydration and Low Temperature

**DOI:** 10.3390/ijms232315050

**Published:** 2022-11-30

**Authors:** Katya Georgieva, Gergana Mihailova, Beatriz Fernández-Marín, Gianpaolo Bertazza, Annalisa Govoni, Miren Irati Arzac, José Manuel Laza, José Luis Vilas, José Ignacio García-Plazaola, Francesca Rapparini

**Affiliations:** 1 Institute of Plant Physiology and Genetics, Bulgarian Academy of Sciences, Acad. G. Bonchev Str., Bl. 21, 1113 Sofia, Bulgaria; 2 Department of Botany, Ecology and Plant Physiology, University of La Laguna (ULL), 38200 Tenerife, Spain; 3 Bioeconomy Institute (IBE), Department of Bio-Agrifood Science (DiSBA), National Research Council (CNR), Via P. Gobetti 101, I-40129 Bologna, Italy; 4 Department of Plant Biology and Ecology, University of the Basque Country (UPV/EHU), Barrio Sarriena s/n, 48940 Bilbao, Spain; 5 Department of Physical Chemistry, University of the Basque Country (UPV/EHU), Barrio Sarriena s/n, 48940 Bilbao, Spain

**Keywords:** resurrection plants, freezing tolerance, desiccation, carbohydrates, fatty acids, protective proteins

## Abstract

Resurrection plants are able to deal with complete dehydration of their leaves and then recover normal metabolic activity after rehydration. Only a few resurrection species are exposed to freezing temperatures in their natural environments, making them interesting models to study the key metabolic adjustments of freezing tolerances. Here, we investigate the effect of cold and freezing temperatures on physiological and biochemical changes in the leaves of *Haberlea rhodopensis* under natural and controlled environmental conditions. Our data shows that leaf water content affects its thermodynamical properties during vitrification under low temperatures. The changes in membrane lipid composition, accumulation of sugars, and synthesis of stress-induced proteins were significantly activated during the adaptation of *H. rhodopensis* to both cold and freezing temperatures. In particular, the freezing tolerance of *H. rhodopensis* relies on a sucrose/hexoses ratio in favor of hexoses during cold acclimation, while there is a shift in favor of sucrose upon exposure to freezing temperatures, especially evident when leaf desiccation is relevant. This pattern was paralleled by an elevated ratio of unsaturated/saturated fatty acids and significant quantitative and compositional changes in stress-induced proteins, namely dehydrins and early light-induced proteins (ELIPs). Taken together, our data indicate that common responses of *H. rhodopensis* plants to low temperature and desiccation involve the accumulation of sugars and upregulation of dehydrins/ELIP protein expression. Further studies on the molecular mechanisms underlying freezing tolerance (genes and genetic regulatory mechanisms) may help breeders to improve the resistance of crop plants.

## 1. Introduction

Low temperatures are one of the most harmful abiotic factors affecting the growth and survival of overwintering plants. These species acclimate to seasonal variations in temperature by adjusting their metabolism during autumn. It is well known that exposure of temperate plants to non-freezing low temperatures increases their freezing tolerance [1]. During cold acclimation, the metabolism is redirected towards the synthesis of cryoprotective molecules, including soluble sugars, sugar alcohols, and other low-molecular-weight compounds (proline, glycine, betaine), as well as towards the synthesis of protective proteins that stabilize enzymes, membranes, and other cellular components [2,3,4]. It has been suggested that the alterations of plasma membrane composition and function are one of the most important mechanisms of plants’ cold tolerance [5]. Under cold conditions, the increased amount of low molecular weight antioxidants (ascorbate and glutathione) and the elevation in the activities of antioxidant enzymes, such as superoxide dismutase, catalase, ascorbate peroxidase, and glutathione reductase, protect plants against the increased formation of reactive oxygen species [6,7]. In addition, the downregulation of photosynthesis in response to low temperatures is considered an adaptive photoprotective strategy that lowers photochemical efficiency to prevent the overexcitation of the photosynthetic apparatus [8,9]. Frost stress induces a further reduction of photosynthesis and, finally, the inhibition of cellular metabolic activities due to the freezing of plant tissues [10]. The freezing of water within plants at subzero temperatures first occurs in the apoplast, which can induce cell dehydration because water is pulled out of the living symplast. This can sometimes dehydrate the cells to less than 10% water content [11]. Thus, during freezing, plants will deal with the risks associated with severe dehydration (unstable cell membranes, reactive oxygen species, photoinhibition), and those unique to freezing (ice crystals), which in the end can cause loss of cell membrane integrity, disruption of structure, and function of cells and tissues [4,12]. Furthermore, the freezing of water in soil reduces water uptake, inducing further cellular dehydration or osmotic stress [13]. Consequently, freezing represents functional and structural perturbations to the cell membranes, plus a higher risk of oxidative damage [14]. Interestingly, both freezing and drought stresses result in the accumulation of similar compatible solutes and hormones, and the existence of cross-acclimation between these two stresses has been suggested [15]; as an example, exposure to drought increases the freezing tolerance of herbaceous and woody species [16,17]. Therefore, it seems that desiccation due to either low temperatures or severe water loss leads to comparable mechanical as well as oxidative risks and that plants utilize similar mechanisms to cope with these extreme stressors [18]. Nevertheless, the double tolerance to freezing and desiccation in tracheophytes has been hardly investigated [19,20,21,22], and many unresolved aspects remain, for instance, how much molecular mobility will be restricted under freezing or low water contents, which can be estimated by assessing glass transition temperatures [18]. Resurrection plants inhabiting cold environments are excellent model organisms to study the keys of the double tolerance to freezing and desiccation in tracheophytes. Studying their metabolic adjustments under natural conditions will enable a deeper understanding of their seasonal acclimation and, thus, of the dynamics of key metabolites involved in one, other, or both extreme tolerances and those involved in either cold acclimation or freezing tolerance.

*Haberlea rhodopensis* is a perennial herbaceous flowering plant belonging to the Gesneriaceae family and represents a tertiary relict on the Balkan Peninsula [23]. It inhabits mainly shaded, northern, chiefly limestone slopes in mountain zones with relatively high humidity. *H*. *rhodopensis* belongs to the group of homoiochlorophyllous resurrection plants that are able to survive desiccation to an air-dry state retaining most of their chlorophyll content [24]. In these plants, the capability to survive extreme intratissular dehydration (i.e., ≤−100 MPa) relies on a complex set of mechanisms to counteract the mechanical and biochemical stress during dehydration and rehydration [25]. In contrast to the other resurrection plants, most of them residing in tropical/subtropical regions [26], the European resurrection angiosperms, including *H. rhodopensis*, must face subzero temperatures during winter in their mountain habitats [21]. Although the ecophysiology of resurrection plants under low temperatures has been understudied, the capability to withstand intraleaf freezing has been evidenced in at least two species: *Ramonda myconi* and *H. rhodopensis* [19,21]. Therefore, in recent years, our research has focused on the mechanisms of freezing tolerance in *H. rhodopensis*, paying special attention to the cold resistance of photosynthetic apparatus [21] and to the role of antioxidant defense in freezing tolerance of this resurrection plant [22]. Our results have shown that cold acclimation of plants does not affect the relative water content (RWC) of leaves and the shape and structure of plastids. However, some decline in photosynthetic activity was observed, which correlated with the decreased proportion of PSII-supercomplexes and the amount of main photosynthetic proteins [21]. The increased content of PSI-LHCII complexes upon cold acclimation suggested an enhancement of energy flow directed to PSI; this state transition may activate the cyclic electron transport, which facilitates the repair of photodamaged PSII. In addition, a significant enhancement of thermal energy dissipation reduced the energy delivery to PSII reaction center and minimized the generation of singlet oxygen [21]. Furthermore, the increased total amount of polyphenols and antioxidant enzymes activity upon cold acclimation contributed to overcoming oxidative stress [22].

Interestingly, our investigations showed that exposure of *H. rhodopensis* to freezing stress (temperatures below −6 °C) induced desiccation of plants and ultrastructural changes in mesophyll cells (at RWC 60%), which are typical for severely desiccated leaves as a result of drought. Freezing stress resulted in a further decline in photosynthetic activity, reduction of the proportion of PSII supercomplexes, significant enhancement in excitation pressure, and increased dissipation of excess excitation energy [21]. The activity of antioxidant enzymes remained high after freezing stress and freezing-induced desiccation, indicating their important role in overcoming oxidative stress [22]. Significant enhancement in flavonoids and anthocyanin content was observed only as a result of freezing-induced desiccation. Thus, after exposure of *H. rhodopensis* to freezing stress, the leaves desiccate very quickly, reaching an air-dry state. This is interpreted as an adaptive strategy to prevent plant damage related to cell destruction under freezing temperatures.

The aim of the present study was to deepen the protective mechanisms during cold acclimation of *H. rhodopensis*, after exposure to freezing stress and in the course of freezing-induced desiccation. For this purpose, we conducted a complete set of analyses aimed at (i) determining the influence of cell dehydration on ice nucleation temperature and on molecular mobility in winter leaves; (ii) characterizing changes in membrane integrity during cold acclimation and freezing stress (by measuring electrolyte leakage, lipid peroxidation, and the content of fatty acids), and (iii) assessing the role of osmolytes (proline, sugars) and protective proteins (dehydrins, ELIPs) in cell preservation during these two processes. The protective mechanisms were studied both under natural environmental conditions (from November until March) and under controlled climatic conditions in a growth chamber.

## 2. Results

### 2.1. Freezing Temperature and Molecular Mobility in Winter Leaves of Haberlea Rhodopensis 

**Figure 1:**
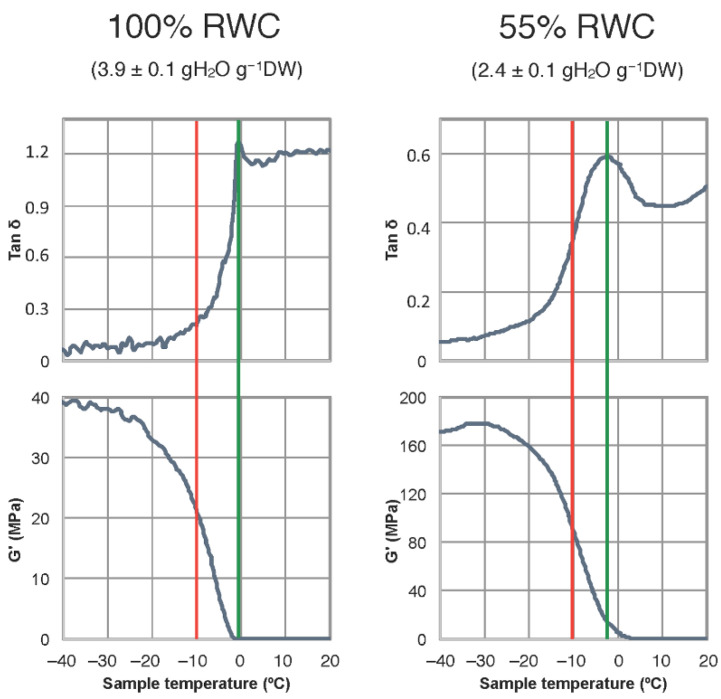
Dynamic mechanical thermal analysis (DMTA) scans of *Haberlea rhodopensis* leaves (dark grey line) at full turgor (100% RWC, (**left**)) or partially dehydrated (55% RWC, (**right**)). The green line highlights the glass transition temperature (Tg), which is identified as a peak in the Tan δ (upper panels). The Red line represents a putative winter temperature in *H. rhodopensis* natural environment. One representative curve from two independent biological replicates is shown for each hydration state.

Both dehydration and freezing events can lower molecular mobility within photosynthetic tissues. To unravel how low temperature may differently affect fully hydrated vs. partially dehydrated leaves of resurrection plants, *H. rhodopensis* leaves at 100% and at 55% RWC were analyzed by DMTA (Figure 1). The average glass transition temperature, Tg ± SE was −0.05 ± 0.53 °C for hydrated and −2.34 ± 0.03 °C for partially dehydrated samples. This indicates higher molecular mobility in partially dehydrated leaves at subzero temperatures. More specifically, the results revealed that at a given winter temperature of, for instance −10 °C (red line), and although molecular mobility is already limited in both types of leaves, enzymatic reactions are more likely to occur in partially dehydrated (55% RWC) than in fully hydrated leaves. In other words, at low temperatures (approximately within the range from +4 °C to −10 °C), a partially dehydrated leaf could show slightly higher metabolic activity than a fully hydrated leaf. This fact is very likely related to the decrease in the freezing temperature of a partially dehydrated sample.

**Figure 2:**
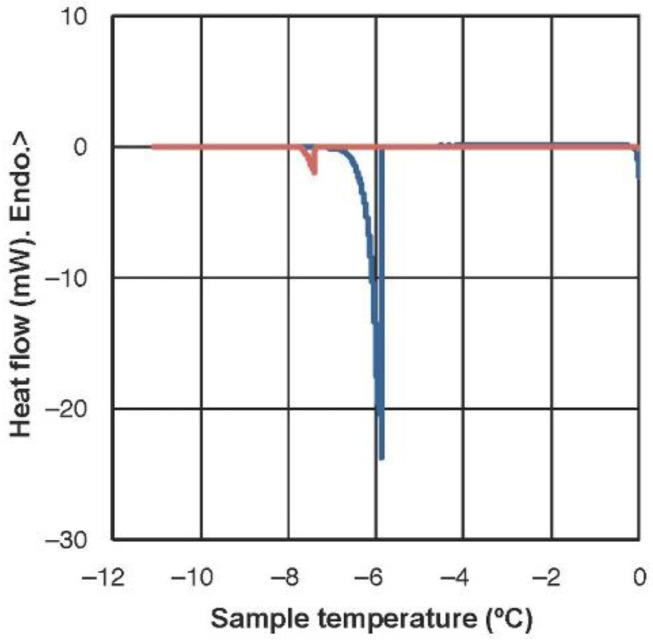
A differential scanning calorimetry (DSC) scan of a hydrated and of a partially dehydrated (RWC = 55%) leaf of *Haberlea rhodopensis*. The scan was recorded at a cooling rate of 0.05 °C min^−1^. One representative scan is shown for each hydration level (blue: hydrated samples, red: dehydrated samples). The average ice nucleation temperature was −5.81 ± 0.30 and −6.62 ± 0.06 °C for leaves at 100% and at 55% of RWC, respectively. Water content was 4.1 ± 0.2 g H_2_O g^−1^ DW and 2.8 ± 0.1 g H_2_O g^−1^ DW for leaves at 100% and 55% of RWC, respectively (average ± SE, *n* = 7).

The lower freezing temperature of partially dehydrated samples is evidenced in Figure 2. The exothermic peaks reveal a major ice nucleation event at −5.81 ± 0.30 °C (average ± SE) in fully hydrated leaves (100% RWC), while it occurred at −6.62 ± 0.06 °C in partially dehydrated leaves (55% RWC).

### 2.2. Changes in Electrolyte Leakage and MDA Content during Cold Acclimation, Freezing Stress and Freezing-Induced Desiccation

#### 2.2.1. Electrolyte Leakage (EL)

**Figure 3:**
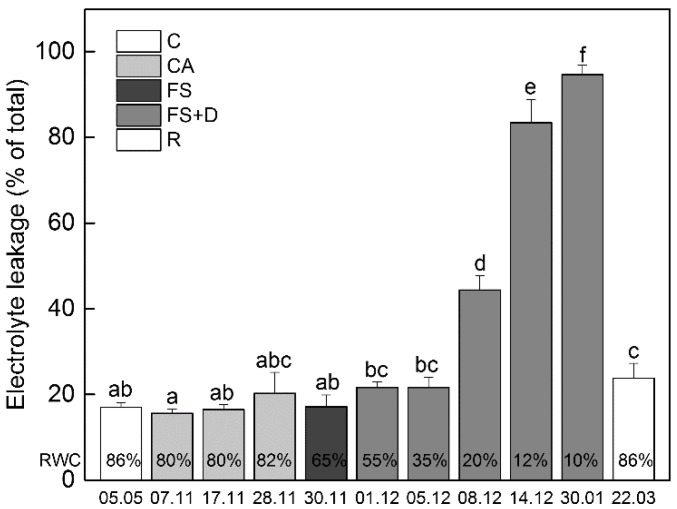
Changes in electrolyte leakage (EL, % of total) during cold acclimation of *Haberlea rhodopensis* (CA, period: 7–28 November), after short-term exposure to freezing stress (FS, −10 °C, 30 November), freezing-induced desiccation (FS + D, period: 1 December–30 January) and after recovery of plants in early spring (R; period: 22 March) under natural ex situ environmental conditions. Percentages at the bottom of the columns show the relative water content (RWC) of leaves at each time point. Data represent the mean of *n* = 6. The same letters within a graph indicate no significant differences assessed by Fisher’s LSD test (*p* ≤ 0.05) after performing ANOVA.

Since cell membranes are a crucial site of freezing injury, changes in the membrane integrity were studied by measuring the extent of electrolyte leakage (Figure 3) during cold acclimation (CA; date 7–28 November), after exposure of plants to overnight freezing stress (FS; −10 °C, date 30 November) and during freezing-induced desiccation (FS + D; date 1 December–30 January). The results showed that EL was not affected significantly during cold acclimation when the average daily and night temperatures were about +10 °C and +2 °C, respectively, and even when the temperature dropped to ca. −10 °C (date 30 November). When freezing stress was concomitant to the decrease of RWC of about 35%, EL gradually increased by about 30% compared to the control (date 5 December). As freezing-induced dehydration proceeded, EL significantly increased up to 83% relative to the control in severely desiccated leaves (12% RWC; date 14 December). Long-term exposure to freezing temperatures (date 30 January) when leaves were severely dehydrated (ca. 10% RWC), led to a further increase in EL. This reached the maximum value of 95% relative to the control. After the rehydration of plants in March, EL sharply decreased and returned back to control values. Similar to ex situ conditions, cold acclimation (2 weeks at +5 °C) and exposure of *H. rhodopensis* to −10 °C (for 12 h) under controlled environmental conditions in a growth chamber did not significantly affect EL.

#### 2.2.2. Malondialdehyde (MDA)

**Figure 4:**
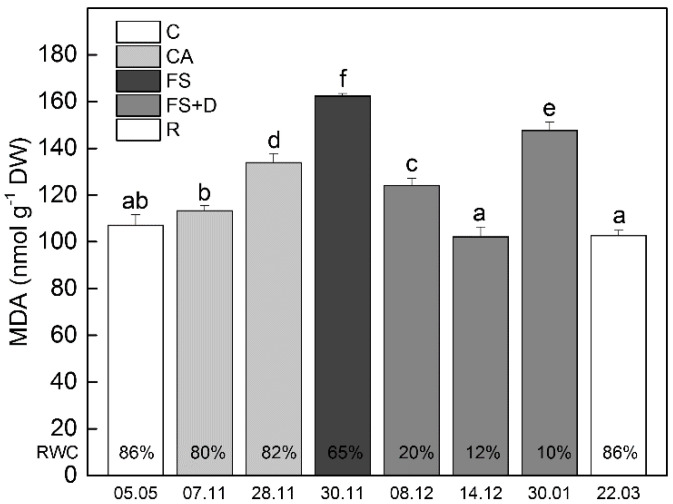
Changes in leaf malondialdehyde (MDA) content (nmol g^−1^ DW) of *Haberlea rhodopensis* after exposure to low temperatures during cold acclimation (CA, period: 7–28 November), freezing stress (FS, −10 °C, 30 November), freezing-induced desiccation (FS + D, period: 8 December–30 January), and after recovery of plants in early spring (R; period: 22 March) under ex situ environmental conditions. The percentages at the bottom of the columns show the relative water content (RWC) of leaves at each time point. Data represent the mean of *n* = 9. The same letters within a graph indicate no significant differences assessed by Fisher’s LSD test (*p* ≤ 0.05) after performing ANOVA.

MDA is a common product of lipid peroxidation and a sensitive marker of oxidative stress. Under natural conditions, MDA content gradually increased during cold acclimation and reached the highest value after overnight exposure to −10 °C (Figure 4; date 30 November). The level of MDA declined as a result of freezing-induced desiccation and reached the value of the control in completely desiccated plants (12% RWC; date 14 December). Like EL, long-term exposure of dry leaves to freezing temperatures induced a further increase in the amount of MDA. These values returned to the control level when plants were rehydrated (date 22 March). Similar to the results obtained under ex situ conditions, when plants were exposed to low temperatures in a climatic chamber, the MDA content slightly enhanced during cold acclimation, but it significantly increased after exposure to −10 °C for 12 h (*p* ≤ 0.05; Figure S1). 

### 2.3. Changes in Fatty Acids Profile under CA, Freezing Stress, and Freezing-Induced Desiccation 

When plants of *H. rhodopensis* were exposed to low temperatures under natural ex situ conditions the fatty acid composition of leaves changed. During cold acclimation (date 28 November) palmitic (16:0; number of carbon:number of double bounds), palmitoleic (16:1) and oleic (18:1) acids decreased significantly, while linolenic (18:3) and behenic (22:0) acids increased (Table 1). During the subsequent overnight freezing stress at −10 °C (date 30 November) palmitic, stearic (18:0), and behenic acids decreased in parallel to an increase in oleic, linolenic, and arachidic (20:0) acids. When freezing stress was concomitant to significant desiccation, the relative contribution of palmitic, palmitoleic, stearic, and oleic acid decreased as compared to the control profile. The most significant enhancement under these conditions was observed in linoleic and especially in linolenic acid, which increased four-fold when RWC reached 12% (date 14 December). The relative amount of the latter fatty acids remained significantly higher than the control when plants regained their water content (date 22 March). As a consequence of these dynamics in fatty acid changes, the ratio of unsaturated/saturated fatty acids in cold-acclimated plants was similar to that found in control plants. However, it increased by 30% (from 0.43 to 0.63) after overnight exposure of plants to −10 °C (date 30 November) due to an increase in the proportion of unsaturated and a decrease in that of saturated fatty acids. The ratio of unsaturated/saturated fatty acids further increased (by 60%; 0.95) in desiccated plants and remained higher than control in rehydrated plants (0.64). Under controlled conditions in a growth chamber, similar changes in the content of unsaturated and saturated fatty acids were observed when plants were exposed to cold acclimation and freezing stress at −10 °C (Table S1). 

### 2.4. Importance of Proline and Carbohydrates for Freezing Tolerance 

#### 2.4.1. Proline

**Figure 5:**
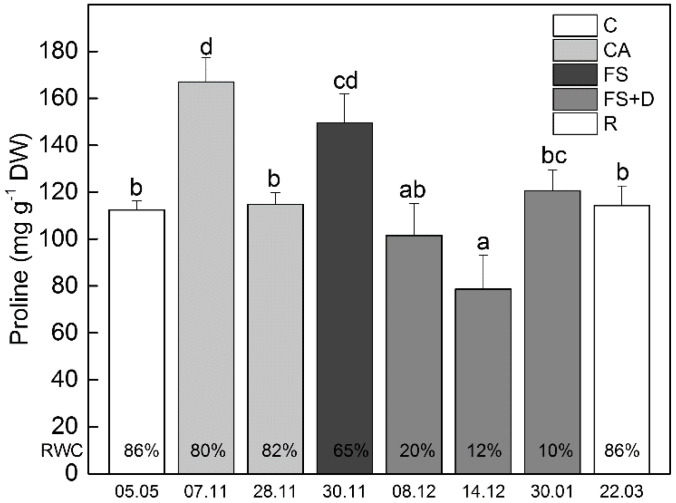
Changes in leaf proline content (mg g^−1^ DW) of *Haberlea rhodopensis* after exposure to low temperatures during cold acclimation (CA, period: 7–28 November), freezing stress (FS, −10 °C, 30 November), freezing-induced desiccation (FS + D, period: 8 December–30 January) and after recovery of plants in early spring (R; period: 22 March) under natural ex situ environmental conditions. Percentages at the bottom of the columns show the relative water content (RWC) of leaves at each time point. Data represent the mean of *n* = 6. The same letters within a graph indicate no significant differences assessed by Fisher’s LSD test (*p* ≤ 0.05) after performing ANOVA.

A significant enhancement in the amount of proline in leaves was observed early during cold acclimation (date 7 November; Figure 5) when mean day and night temperatures for two weeks before sampling was about +13 °C and +4 °C, respectively. The following overnight freezing stress (date 30 November; a temperature of ca. −10 °C) also resulted in an increased proline content. On the contrary, the subsequent freezing-induced desiccation led to a decrease in its amount. Under controlled conditions in the climatic chamber, the magnitude of proline response to cold temperatures was similar to that observed ex situ, while the increase of this osmolyte after 12 h exposure to −10 °C was lower compared to that observed under natural freezing conditions when leaf dehydration started (Figure S2).

#### 2.4.2. Carbohydrates

The soluble carbohydrates found in detectable levels in leaves of *H. rhodopensis* were the hexoses glucose, fructose, and galactose, the disaccharide sucrose, the trisaccharide raffinose, galactose, and the sugar alcohol inositol. Sorbitol and trehalose were detected only at trace concentrations. Most of these sugars have been previously detected in this resurrection species [27,28,29,30]. Within the soluble sugar pool, the disaccharide sucrose and the trisaccharide raffinose were the most abundant carbohydrates (ca. 12 and 6 mg g^−1^ DW in leaves of control plants), followed by the monosaccharides glucose and fructose (ca. 1–2 mg g^−1^ DW; Figure S3). Galactose and inositol ranged around 1–2 mg g^−1^ DW over the entire study (Figure S4). 

Other organic metabolites (namely, malic and citric acid) that are important intermediates of the tricarboxylic acid metabolism were detected in the order of a few milligrams on a dry weight basis (Figure S4).

The majority of the identified soluble carbohydrates and carboxylic acids accumulated in response to low temperatures over the study period (7 November–14 December; Table 2; Figure S3). When plants experienced back temperature conditions of almost +20 °C (date 23 March), the abundance of these metabolites returned back to almost control levels (Figure S3).

The extent of sugar and carboxylic acid accumulation differed depending on the individual compound and on the exposure conditions (time and temperature). The most pronounced enhancements were observed for sucrose and raffinose, reaching a maximum (absolute value of 185 mg g^−1^ DW and 101 mg g^−1^ DW, respectively; ca. 16-fold control values in both sugars; Figure S3; Table 2) in severely desiccated plants (date 14 December). Generally, glucose and fructose accumulated significantly, but to a lesser extent exhibiting the highest concentration (9 mg g^−1^ DW and 14 mg g^−1^ DW, respectively; ca. eight-fold the control values in both sugars) at an earlier time point (date 8 December) compared to that of sucrose and raffinose. Interestingly, in the middle of December, the peak of sucrose and raffinose accumulation was concomitant to a depletion of monosaccharides, which consequently accounted for only 2–3% of total sugar content. Galactose and inositol contents doubled early during CA, while they slightly increased or almost leveled off, respectively, as freezing stress progressed (Table 2). 

**Figure 6:**
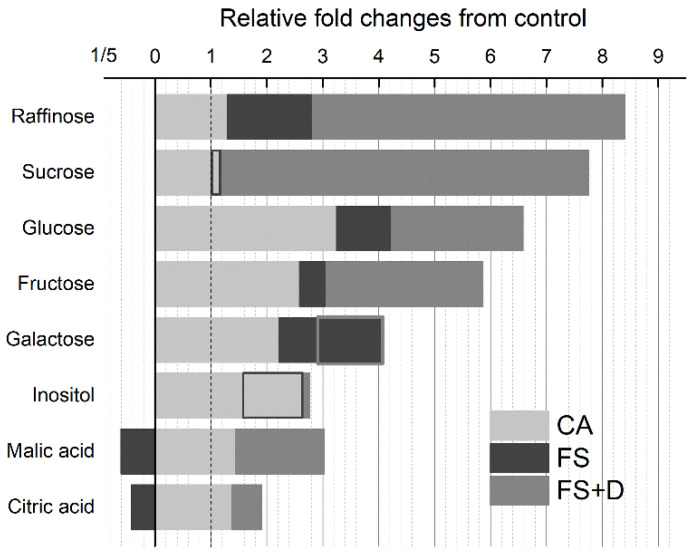
Changes in the mean concentration of carbohydrates and organic acids measured in leaves of *Haberlea rhodopensis* from the control under ex situ environmental conditions. Light grey bars indicate changes between control (May) and cold acclimation (CA; period 7–28 November). Dark grey bars indicate changes from CA to short-term exposure to freezing temperatures (FS; −10 °C, 30 November). Grey bars indicate changes between FS and longer-term exposure to freezing temperatures when significant desiccation occurred (FS + D; period: 5–14 December). The bars for compounds for which the trend between one phase was opposed to that observed during the precedent transition have only the grey outlines.

We calculated fold changes in the mean concentration of carbohydrates and organic acids from the control to investigate the contribution of individual compounds to the different phases of natural exposure of *H. rhodopensis* to low temperatures (Figure 6). During the acclimation phase, the monosaccharides (namely, glucose, fructose, and galactose), as well as inositol, exhibited a relatively higher enhancement (ca. two-three-fold) compared to the other metabolites. On the contrary, most of the relatively large accumulation of sucrose and raffinose (ca. eight-ninefold relative to respective controls) occurred during the transition to the subsequent phase of freezing stress (FS and FS + D; 30 November–14 December). However, differently from sucrose, raffinose showed an early twofold increase during the transition from CA to overnight exposure to −10 °C (FS; 30 November). Similar to sucrose and raffinose, organic acids showed consistent changes when plants experienced freezing stress (Table 1). Exposure to overnight −10 °C resulted in a depletion of both malic and citric acids. By contrast, subsequent freezing-induced desiccation determined an increase in these acids (14 December; Figure 6). 

Overall, the results obtained under temperature-controlled conditions (Figure S5) confirmed those observed under natural exposure to low temperatures. After two weeks of CA at constant +5 °C the magnitude of glucose and fructose increases were generally comparable with those observed under ex situ CA conditions (Figure S6). Conversely, the accumulation of sucrose and raffinose during cold exposure in the climatic chamber differs from that observed when plants were exposed to CA under environmental conditions. Constant exposure to cold temperatures and light/dark cycles in the climatic chamber may result in different modulations of carbohydrate metabolism compared to relatively variable environmental conditions. The transition from CA to freezing stress (12 h treatment at −10 °C; FS) induced relative increases in the majority of sugars (i.e., glucose, fructose, sucrose, and raffinose) similar to those measured under natural ex situ conditions when plants experienced overnight exposure to ca. −10 °C. Changes in organic acids in the climatic growth chamber were irrelevant under both CA and FS. 

**Figure 7:**
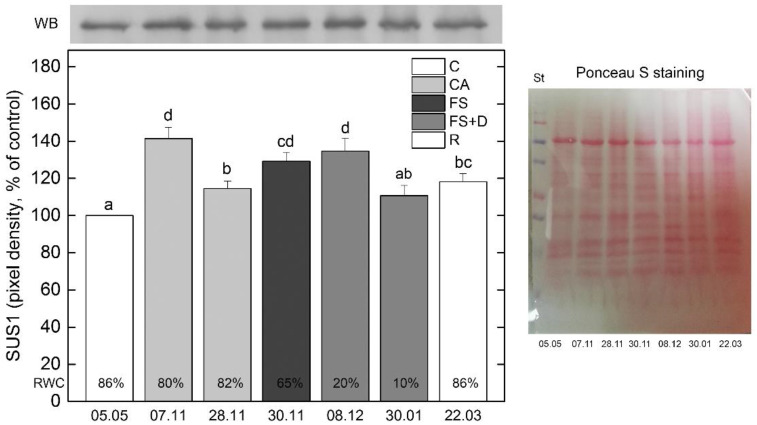
Changes in the expression of Sucrose synthase 1 (SUS1) protein in leaves of *Haberlea rhodopensis* after exposure to low temperatures during cold acclimation (CA, period: 7–28 November) freezing stress (FS, −10 °C, 30 November), freezing-induced desiccation (FS + D, period: 8 December–30 January) and after recovery of plants in early spring (R; period: 22 March) under natural ex situ environmental conditions. A representative Western blot is presented in the graph. 30 µg protein was applied per lane. Percentages at the bottom of the columns show the relative water content (RWC) of plants at each sampling point. The same letters within a graph indicate no significant differences assessed by Fisher’s LSD test (*p* ≤ 0.05) after performing ANOVA. Ponceau S staining of the membrane after blotting is presented on the right. SUS1 values are normalized according to Ponceau S staining. St: Precision Plus Dual Color Protein™ Prestained Standards (Bio-Rad, Hercules, CA, USA).

Sucrose synthase 1 (SUS1) is a key enzyme of sucrose metabolism catalyzing the reversible conversion of sucrose and a nucleoside diphosphate into the corresponding nucleoside diphosphate-glucose and fructose. Considering the literature data showing the involvement of SUS1 in response to osmotic stress [31,32], we monitored its expression by immunoblot analysis using specific antibodies raised against SUS1. SUS1 appeared as a single protein band, having a molecular mass of approximately 90 kDa (Figure 7). Western-blot analysis revealed the increased content of SUS1 in leaves of *H. rhodopensis* during cold acclimation, overnight freezing stress of −10 °C and freezing-induced desiccation under ex situ environmental conditions.

### 2.5. The Role of Protective Proteins for Freezing Tolerance of H. Rhodopensis

#### 2.5.1. Dehydrins

**Figure 8:**
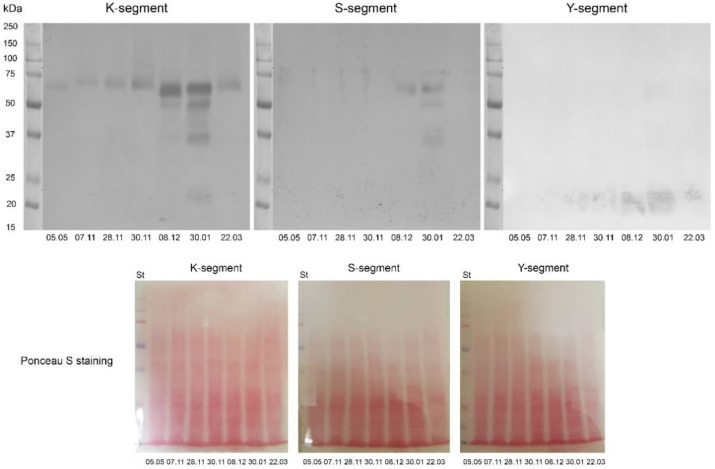
Representative Western blots of dehydrins (**top**) in leaves of *Haberlea rhodopensis* after exposure to low temperatures during cold acclimation (CA, period: 7–28 November), freezing stress (FS, 30 November, −10 °C), freezing-induced desiccation (FS + D, period: 8 December–30 January) and after recovery of plants in early spring (R; period: 22 March) under natural ex situ environmental conditions detected by Western blot using antibodies against the conserved K-, S- and Y-segment of the proteins. 30 µg protein was applied per lane. Ponceau S staining of the membranes after blotting is presented below the Western blots. St: Precision Plus Dual Color Protein™ Prestained Standards (Bio-Rad, Hercules, CA, USA).

Dehydrins are hydrophilic proteins belonging to the family of late embryogenesis abundant (LEA) proteins, known as LEA2 group proteins. We monitored the expression of dehydrins during cold acclimation, freezing stress and freezing-induced desiccation of *H. rhodopensis* under ex situ environmental conditions by Western blot using antibodies raised against the conserved K-, S-, and Y-segment of the proteins (Figure 8). Immunoblot analysis with K-antibody visualized at least 10 different bands with apparent molecular weights of around 17–80 kDa (70, 60–65, 50, 40–42, 30, 27, 25, 22, and 17 kDa). The band at approximately 60–65 kDa was present in all investigated samples and was the only band detected in control plants (date 5 May; Figure 8). During cold acclimation (date 7 November and 28 November) and after exposure of plants to freezing stress (date 30 November) the molecular weight of this band was shifted and the protein migrated to a higher molecular weight. In severely desiccated plants, the protein migrated to lower molecular weight (date 8 December and 30 January– 20% and 10% RWC). Cold acclimation and freezing-induced desiccation enhanced the intensity of this band. Faint signals around 80, 22 (double band), and 17 kDa were visible in all of the investigated samples except the control. Cold acclimation and freezing-induced desiccation enhanced the intensity of 22 kDa band. In fact, exposure of plants to about −10 °C did not induce significant changes in the expression of dehydrins. However, freezing-induced desiccation (data 8 December and 30 January) induced the expression of new dehydrins with molecular weight between 25–50 kDa. After the recovery of plants (date 22 March), the content of newly synthesized dehydrins during freezing-induced desiccation (25–50 kDa) sharply decreased, and the detected bands were very weak. Cross-detection with antibodies against K- and S-segment revealed the presence of the S-segment in the proteins with an apparent molecular weight 70 kDa in all samples. Signals were also visible in 60–65 kDa proteins in desiccated samples (date 8 December and 30 January) and 50, 40–42 (double band) and 30 kDa proteins at 30 January. No signals were detected in samples from recovered plants (date 22 March). Cross-detection with antibodies against K- and Y-segment showed the appearance of Y-segment in low molecular weight proteins (22 kDa, 17 kDa) in all samples, except control plants where those proteins were not detected with K- and S-antibodies. The Y-segment was also visible in 50, 40–42, and 30 kDa proteins (faint signals) in desiccated plants exposed to long-term freezing temperatures (date 30 January).

Immunoblot analysis of samples from cold-acclimated plants and those exposed to −10 °C in a growth chamber showed similar results (Figure S7). However, it seems that *H. rhodopensis* experienced stronger freezing stress after cold acclimation for 2 weeks at +5 °C under controlled conditions than plants exposed to ex situ conditions, resulting in enhanced expression of dehydrins. The use of an antibody against the conserved S-segment of dehydrins revealed the presence of 50 kDa signal in control and CA plants. This 50 kDa signal was not visible in membranes probed with K-segment antibody, probably because of the different amino acid sequences of the two antibodies used. Cross-detection with antibodies against K- and Y-segment showed the appearance of Y-segment in low molecular weight proteins (22 kDa) in all samples. One band could be seen in controls (C), whereas two bands were visible in the other three samples (CA, FS, R).

#### 2.5.2. ELIPs

**Figure 9:**
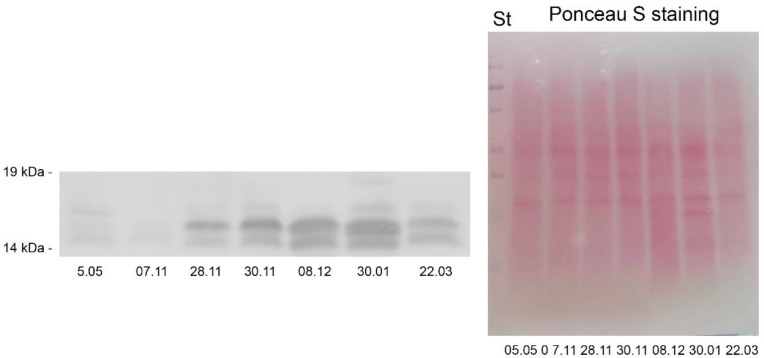
Representative Western blots of ELIPs (**left**) in leaves of *Haberlea rhodopensis* after exposure to low temperatures during cold acclimation (CA, period: 7–28 November), freezing stress (FS, 30 November, −10 °C), freezing-induced desiccation (FS + D, period: 8 December–30 January) and after recovery of plants in early spring (R; period: 22 March) under natural ex situ environmental conditions. 30 µg protein was applied per lane. The Ponceau S staining (**right**) of the membrane after blotting is presented on the right. St: Precision Plus Dual Color Protein™ Prestained Standards (Bio-Rad, Hercules, CA, USA).

The early light-induced proteins (ELIPs) are stress-induced proteins with proposed photoprotective function and are related to LHC a/b-binding proteins [33]. We monitored their expression during cold acclimation and freezing-induced desiccation of *H. rhodopensis* under ex situ environmental conditions by Western blot using antibodies raised against ELIPs. After SDS-PAGE separation of isolated total leaf proteins and Western blot, several bands with apparent molecular weight around 14–19 kDa were detected in the investigated samples, but it seems that ELIPs were differentially expressed, quantitatively and qualitatively (Figure 9). Two main bands were observed in almost all the samples (upper and lower), while the third band appeared (between the main two, but close to the upper one) when the RWC of the plants dropped to 20% and 10%. In control plants (date 5 May) and samples collected at the beginning of November (7 November) very small amount (traces) of ELIPs could be seen. The protein expression of ELIPs gradually increased in CA plants after exposure to freezing stress and freezing-induced desiccation (from 28 November to 30 January). The increase was accentuated in the severely dehydrated plants (8 December and 30 January), in which the third (middle) band appeared. In these plants, the amount of ELIPs was three to five times higher than in plants exposed to −10 °C (date 30 November). After the recovery of *H. rhodopensis* plants (date 22 March), the expression of ELIPs decreased, the middle (third) band disappeared, but the amount of the proteins was still much higher than in control plants (5 May). In a parallel experiment under controlled conditions, we obtained similar results about ELIPs—very faint bands in control plants with the following increase of the protein expression after 2 weeks at 5 °C and 12 h at −10 °C (Figure S8). Similar to dehydrins, exposure to −10 °C in a growth chamber resulted in enhanced expression of ELIPs compared to ex situ conditions.

### 2.6. Correlation Patterns among Physiological and Biochemical Parameters

**Figure 10:**
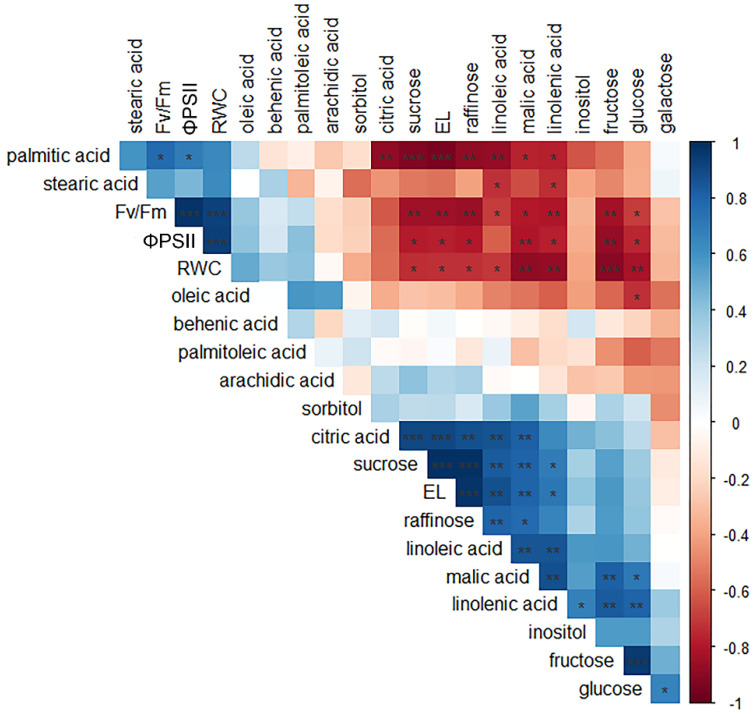
Correlation matrix for leaf relative water content (RWC), electrolyte leakage (EL), photochemical parameters (ΦPSII and Fv/Fm), and biochemical variables (sugar and fatty acids content) measured at midday in leaves of *Haberlea rhodopensis* when exposed from control to low temperatures. The color scale ranges from red to blue according to the maximum (−1) and minimum (+1) Pearson’s correlation coefficient, respectively. Significance correlations are indicated as: * *p ≤* 0.05; ** *p ≤* 0.01; *** *p ≤* 0.001.

A correlation analysis was conducted to better explore the relationships between physiological and some biochemical parameters (i.e., soluble sugars, carboxylic acids, and fatty acids) examined under natural ex situ conditions (Figure 10). Overall, seven metabolites, including fructose, glucose, sucrose, linolenic acid, linoleic acid, and malic acid showed significant correlations with the examined physiological parameters. In particular, the parameter EL showed significant positive correlations with sucrose and raffinose (*r* > 0.98, *p* < 0.001 for both sugars). RWC correlated negatively with the content of four sugars (i.e., glucose, fructose, sucrose, and raffinose; r values ranging between −0.73 and −0.90; 0.05 > *p* < 0.001), of fatty acids (i.e., linolenic and linoleic acid; *r* = −0.87, *p* < 0.01 and −0.70 *p* < 0.05, respectively), and of organic acids (i.e., malic acid; *r* = −0.89, *p* > 0.01). Interestingly, all the above metabolites also showed strong and significant negative correlations with photochemistry parameters (i.e., ΦPSII and Fv/Fm; unpublished data), especially evident for the four sugars (*r* values ranging from 0.70 to 088; 0.05 > *p* < 0.01). Among sugars, a strong positive correlation was observed between sucrose and raffinose (*r* = 0.98, *p* > 0.001) and between fructose and glucose (*r* = 0.95, *p* < 0.001).

## 3. Discussion

In the current study, we extended our understanding of the protective mechanisms that may be involved in the ability of the resurrection *Haberlea rhodopensis* to cold acclimate during autumn and to survive freezing temperatures in winter.

### 3.1. Changes in Membrane Lipids Composition Contribute to Freezing Tolerance

The protection and maintenance of cell membranes is a key factor in the plant survival to low temperatures as they are vulnerable to freezing-induced injury [6,14,34]. In light of our results, *H. rhodopensis* has efficient preservation of cell membrane integrity under low temperatures. This was supported by the lack of significant changes in electrolyte leakage when plants of *H. rhodopensis* were acclimated to chilling temperatures or were exposed to brief freezing stress (ca. −10 °C, 12 h). By contrast, the pronounced enhancement in EL when prolonged exposure to freezing temperatures resulted in severe leaf dehydration (RWC < 20%) resembles the overall pattern of EL changes that have been previously observed in this resurrection species under desiccation [35]. The fast diminution of EL after rehydration of freezing-desiccated plants further supports the relevance of the processes that maintain membrane stability as a survival mechanism [36].

Our previous results on the same plants used in the present experiment showed that a high leaf antioxidant activity contributes to the maintenance of membrane integrity by scavenging free radicals [22]. In addition, data from the current experiment suggest that the ability of *H. rhodopensis* to tolerate freezing temperatures after cold acclimation could rely on changes in membrane lipids and, in particular, on the elevated ratio of unsaturated/saturated fatty acids observed during freezing exposure, especially when severe leaf dehydration occurred. Indeed, a high relative proportion of unsaturated fatty acids is considered crucial to maintaining membrane fluidity and cellular function when plants are exposed to low temperatures [2,6]. Accordingly, an increasing level of lipid unsaturation at decreasing temperatures has been observed in the leaves of numerous herbaceous and woody species [34,37,38], including resurrection species [39,40].

### 3.2. The Role of Proline and Soluble Sugars in Freezing Tolerance

Differently, the observed transient and moderate changes of leaf proline suggest its minor role for freezing tolerance of *H. rhodopensis,* although it may play a role in other resurrection species such as *R. myconi* [20]. The transient leaf accumulation of proline at the early stages of chilling and freezing might be related to a rapid osmotic adjustment. However, the subsequent decline of this metabolite under prolonged low-temperature conditions, particularly evident in fully freezing-desiccated plants, could be associated with the concomitant elevated accumulation of alternative protective compounds.

Among the compounds isolated from leaves of *H. rhodopensis*, soluble sugars were distinguished for being the most responsive metabolites to low temperatures with significant increases in glucose, fructose, sucrose, and raffinose, and in other carbohydrates such as galactose and inositol. Although in resurrection species, the accumulation of sugars has been proposed as an important mechanism to cope with desiccation [41], the response of carbohydrate metabolism to low temperatures has been poorly explored. Sugar accumulation under low temperatures has been long reported in a wide variety of species [42], while in the resurrection angiosperm species it has been shown only in response to chilling in *H. rhodopensis* [43] and to freezing in *R. myconi* [20].

The high correlations between electrolyte leakage and the accumulation of the predominant soluble sugars (i.e., sucrose and raffinose) in *H. rhodopensis* are consistent with the multifunctional role of soluble sugars in the acquisition of tolerance to low temperatures [44,45]. By acting as osmoprotectants, they limit freezing-induced dehydration, prevent ice nucleation inside the cell, and stabilize cellular membranes and protein structure [46,47]. In addition, sucrose and raffinose are involved in classic antioxidative mechanisms, as well as in direct ROS quenching, especially when they are present at high concentrations [48]. Since, in chloroplasts, both sucrose and raffinose have been recognized to protect the photosystem II during chilling [49] and freezing conditions [50], the observed high correlation between these sugars and PSII activity further supports the functional relevance of the accumulation of these sugars for the survival of *H. rhodopensis* under low-temperature conditions.

Although tolerance of *H. rhodopensis* to low temperatures may rely on the increased content of soluble sugars, our results highlight those dynamics and interdependency of carbohydrate changes may be crucial for cold acclimation and for freezing stress tolerance. Different timing and extent of carbohydrate accumulation were evident in this resurrection species under decreasing low temperatures. During cold acclimation, a rapid increase of soluble sugars occurred mainly in the form of hexoses (i.e., glucose, fructose, and galactose). In this phase, they may serve in osmotic adjustment to maintain leaf water content. When fully acclimated plants were exposed to freezing temperatures, carbohydrate metabolism was shifted to an increased formation of sucrose and raffinose, a pattern consistent with their major role in cryoprotection in addition to other protective functions against freezing-induced desiccation. In other resurrection species, a similar pattern of early and late changes in carbohydrate content has been observed in response to progressive loss of water [41,51,52], but never under low-temperature conditions.

If freezing-induced desiccation is an important survival strategy of *H. rhodopensis* to avoid irreversible damage due to ice crystals formation and survive freezing temperatures during winter, the accumulated soluble sugars can facilitate vitrification, i.e., the formation of a glassy state that slows molecular motion and reduces the deleterious chemical reactions [44,47]. In particular, the elevated concentration of sugars can lower the temperature at which ice formation is initiated in plant tissues [44]. Data presented here, showing lowered glass transition temperatures at partially dehydrated leaves (RWC 55%) when compared to fully hydrated leaves, agree with this and with the fact that the bulk of “freezable water” will be reduced in non-turgor leaves [14]. Taken together, these findings suggest that in cold-acclimated plants of *H. rhodopensis* key functional mechanisms may protect against subsequent freezing damage: a lowering of ice-forming temperature may prevent physical damages directly induced by ice-crystals, while the observed enhanced molecular mobility could warranty longer metabolic activity for protection and repairing mechanism with progressive freezing conditions and during post-thawing recovery. Interestingly, the ice nucleation temperature of turgor leaves was lower in *H. rhodopensis* than in the other mountain resurrection angiosperm *R. myconi* [19]. This suggests that despite these species sharing similar mechanisms for surviving low temperatures, they might be characterized by distinct constitutive components that confer tolerance to low temperatures.

Data presented here on carbohydrate dynamics in response to freezing temperatures in *H. rhodopensis* are consistent with the proposed multi-stress tolerance of the Gesneriaceae to both desiccation and low temperatures [20]. Indeed, similarities between the response of sucrose to freezing temperature and that to water deficit are evident in this resurrection species: sucrose accumulation started when dehydration fell below 55% both under freezing conditions or water deficit alone [30]. When *H. rhodopensis* plants are exposed to freezing conditions, the enhancement of sucrose content appears to be initiated mainly by desiccation signals. On the other hand, raffinose accumulation commences earlier, compared to sucrose, appearing upon exposure to a short period of freezing temperature (ca. −10 °C; overnight) when leaf water content was still moderate (RWC above 55%). Considering that in our previous study on *H. rhodopensis* raffinose content was not affected by desiccation [30], our current findings are consistent with the accumulation of raffinose initiated by sensing of freezing *per se*. Similarly, in the resurrection species, *R. myconi* the enhancement of the soluble sugars pool was found to be a specific response to low temperatures [20]. It is worth highlighting that although the accumulation of sucrose is a common response to desiccation in many angiosperm resurrection plants [27,29,43,52], the enhanced increase of the raffinose pool is not always found [41]. Like other predominant members of the raffinose family oligosaccharides, raffinose is recognized as a crucial protective component of plant tolerance to low temperatures [53]. In previously published studies, the ability of *H. rhodopensis* to tolerate different abiotic stress conditions has been linked to a specific function of raffinose [28,29]. The high constitutive levels of raffinose in resurrections plants of Gesneriaceae, including *H. rhodopensis*, have been considered a relevant pre-adaptation trait for desiccation tolerance [27,29,30]. Our present findings show that raffinose represents not only an important constitutive resource of this resurrection species for tolerating low temperatures but also a highly inducible component (from ca. 1% up to more than 10% of the leaf DW over the time course of low temperatures) to counteract freezing-induced damages. The induction of raffinose formation might contribute to preventing the crystallization of sugars, in particular of sucrose, and facilitates cytoplasmatic glass formation [54,55].

In addition to the dynamics of the soluble sugar pools, their interdependencies during freezing-induced desiccation provide further interesting insights into the multi-stress tolerance of *H. rhodopensis*. When freezing stress was concomitant to severe dehydration, the maximum increase of sucrose coincided with a concomitant depletion of the hexoses (fructose and glucose) that had accumulated up to this point. The sucrose–hexose interconversion, probably contributing to the increased sucrose content, has been previously observed in *H. rhodopensis* [27,28,29,56] and other resurrection angiosperm species [40,41,55] in response to water deficit but not to low temperatures. Taken together, our findings on *H. rhodopensis* suggest that a redirection of soluble carbohydrate metabolism in favor of sucrose and raffinose is involved in developing tolerance not only to desiccation but also to low temperatures. This mechanism has been considered relevant in the model species Arabidopsis during its adaptation to low temperatures [57].

The accumulation of soluble sugars and a decrease in leaf osmotic potential may be responsible for the observed upregulation of sucrose synthase 1 (SUS1) [31,58]. On the other hand, the enhanced content of this enzyme participating in sucrose cycling suggests that other carbon sources of sucrose should be considered to achieve the accumulation of this sugar. The contribution of starch hydrolysis in the prominent accumulation of sucrose during freezing stress is supported by qualitative detection of starch breakdown in leaves of the same plants of the current study [21].

Among other potential metabolic changes, the involvement of organic acids (i.e., malic and citric acid), which are important metabolites of the tricarboxylic acid cycle, in the acquisition of freezing tolerance of *H. rhodopensis* is supported by their high correlation with both EL and PSII activity. Leaf changes in the content of these organic acids were detected in the mountain angiosperm resurrection species *R. myconi* after exposure to low temperatures in natural winter conditions [20].

### 3.3. The Protective Role of Dehydrins and ELIPs during Freezing Stress 

Our findings on profiling of dehydrins and ELIPs, in *H. rhodopensis* reveal that in response to low temperatures, a general accumulation of these stress-induced proteins is evident, indicating a relevant contribution to the capacity of this resurrection species to cold acclimate and tolerate freezing temperatures. During freezing-induced desiccation, a more distinct compositional change is evident, suggesting the specific cellular functions of individual proteins may be relevant depending on the time and intensity of low-temperature exposure.

The accumulation of dehydrins is induced by various environmental stresses, including low temperatures and drought [37,59,60], and has been associated with freezing tolerance [61]. Indeed, these proteins are assumed to possess cryoprotective and antifreeze activity as well as prevent denaturation of cellular components under desiccation [62]. The observed enhancement of dehydrins (60–65 kDa and 22 kDa), together with the appearance of faint signals in response to cold and freezing temperatures, is consistent with a similar cold-induced pattern in other plant species [63,64]. The specific accumulation of dehydrins containing the K- and Y-segments in their structure during cold acclimation has been previously observed in herbaceous species [65]. The freezing-induced increase of dehydrins, together with the expression of new dehydrins (25–50 kDa) in freezing-desiccated plants, reflects a higher requirement of dehydrins for the protection of *H. rhodopensis* in our experimental conditions. In several resurrection plants, a consistent increase of dehydrin expression has also been reported in response to dehydration [66,67,68], suggesting that in *H. rhodopensis* the accumulation of dehydrins can be considered a shared mechanism of tolerance to both low temperatures and desiccation.

In addition to dehydrins, the increased protein expression of ELIPs in leaves of *H. rhodopensis* during cold acclimation and after exposure to freezing temperatures is indicative of an adaptive mechanism to winter low-temperature conditions as reported in several overwintering species [8,69,70,71]. Similar to dehydrins, the increase of ELIPs was evident, especially during freezing-induced dehydration, reaching a maximum in severely desiccated plants, where a new protein band appeared. It is proposed that ELIPs fulfill a protective role of the photosynthetic apparatus against photoinhibition within the thylakoids by preventing the formation of free radicals and/or by acting as sinks for excitation energy [72]. In the resurrection *Craterostigma plantagineum* the accumulation of 22 kDa ELIP-like desiccation-induced protein in the thylakoid membranes have been proposed to play a protective role against photoinhibition [73]. The transcriptomic analysis revealed an increased abundance of ELIP genes in response to desiccation in the leaves of several resurrection species [74,75,76,77], including *H. rhodopensis* [56]. As in the case of dehydrins, these results indicate that common responses of *H. rhodopensis* plants to low temperature and desiccation involve the upregulation of ELIP protein expression.

## 4. Materials and Methods

### 4.1. Plant Material and Temperature Treatments

*Haberea rhodopensis* Friv. tufts were initially collected from the Rhodope Mountains, where they grow in deeply shaded rock-crevice habitats at a light intensity of approximately 25 µmol m^−2^ s^−1^, and further cultivated under ex situ conditions. Two different experimental systems were applied to evaluate the responses of *H. rhodopensis* to low temperatures: in one experiment, plants were exposed to natural annual low temperatures occurring during the autumn–winter period; in a second experiment, plants were subjected to decreasing temperatures in a growth chamber. The realization of both experiments allowed us to confirm and better explain the obtained results during natural cold acclimation and/or during short-term exposure to freezing temperatures of about −10 °C. Because leaf desiccation occurred only under natural freezing conditions, the experimental design allows distinguishing the effects of freezing stress alone and freeze-induced desiccation. For experiment 1, low-temperature responses of *H. rhodopensis* were evaluated in plants growing outdoors under natural climatic conditions, where they were exposed to low temperatures that occurred during autumn–winter from November 2016 to January 2017. Minimum and maximum daily ambient temperatures are reported in Mihailova et al. [21] and are summarized in Table S2, where the specific sampling dates are also reported. At each time point, leaves were collected from at least ten different tufts (with 4–7 individual plants in a tuft) to have mean samples. Over the autumn–winter period, the average daily light intensity under natural conditions ranged between 30 and 60 μmol m^−2^ s^−1^ PPFD. The samples were taken at different time points from November 2016 to March 2017 in order to monitor their physiological and biochemical responses to cold acclimation and freezing stress, as well as during recovery from low temperatures. Control (C) samples were collected in May.

The experiment consisted of the following time points of low-temperature exposure:

(1) Cold acclimation (CA): plants were sampled during gradual acclimation to low daily mean temperatures ranging from +1 °C to +7 °C (dates 7–28 November; CA).
(2) Freezing stress (FS): after an overnight freezing wave exposure to ca. −10 °C, resulting in a slight desiccation of the leaves (ca. 25% reduction in RWC compared to the control) on the sampling day (date 30 November).
(3) Freezing stress + desiccation (FS + D): plants were sampled during long-term exposure to freezing temperatures when significant desiccation of the leaves occurred (RWC ranging from 10 to 20%) from the beginning of December 2016 (date 1 December) till the end of January (date 30 January); During December, nighttime temperatures were negative, and the average daily temperature was ca. +5 °C, while in January for more than 20 days minimum temperatures reached values in the range of −8 °C to −17 °C. As a consequence, sampling on date 30 January is representative of long-term exposure to freezing temperatures compared to December samplings.
(4) Recovery (R): plants were sampled in March 2017 as representative of plant recovery from freezing-induced desiccation.

For experiment 2, plants were kept outdoors until October and then transferred to a climatic chamber FytoScope FS 130 (Photon Systems Instruments, Drasov, Czech Republic), where they were grown for two weeks under conditions of +20/+15 °C day/night temperature, 12 h photoperiod and 30 μmol m^−2^ s^−1^ photosynthetic photon flux density (PPFD). Then plants were exposed to a temperature of +5 °C, and light intensity of 30 μmol m^−2^ s^−1^ with a 12 h photoperiod for 2 weeks, accordingly to the natural cooling rate [21]. After acclimation to cold temperatures, plants were exposed in darkness to short-term freezing stress by lowering the temperature at a rate of 1 °C/h, until a minimum of −10 °C was reached and maintained for 12 h. Subsequently, the growth chamber temperature was gradually increased up to 20/15 °C and maintained for 24 h under the conditions of light intensity of 30 μmol m^−2^ s^−1^ with a 12 h photoperiod. This experiment was repeated twice. At each time point, leaves were collected from at least six different plants for each experiment.

For both experiments, fully expanded leaves from the middle of the rosettes were collected at midday and used for the analysis. The phenology and physiological state of the investigated plants under both natural and controlled environmental conditions are fully described in Mihailova et al. [21].

### 4.2. Relative Water Content (RWC)

The RWC of *H. rhodopensis* leaves was determined gravimetrically by weighing them before and after oven-drying at 80 °C to a constant mass and expressed as a percentage of water content in dehydrated tissue compared to water-saturated tissues, using the equation:

RWC (%) = (FM − DM)/(TM − DM) × 100, where FM − fresh mass, DM − dry mass, and TM − turgid mass. TM was measured on leaves maintained for 12–16 h at 4 °C in the dark floating on water.

### 4.3. Dynamic Mechanical Thermal Analysis (DMTA)

Fully hydrated or partially desiccated leaf discs (ø 12 mm) from control leaves of *H. rhodopensis* were used to determine molecular mobility. Two discs obtained from the same leaf were used for each measurement, and three biological replicates were assessed. Measurements were conducted with a DMA/SDTA861e mechanical thermal analyzer (Mettler Toledo, Greifensee, Switzerland) in the shear mode following the method described in Fernández-Marín et al. [19,78]. Briefly: tests were carried out in the dynamic mode from −50 to +150 °C at a heating rate of 2 °C min^−1^. Frequencies used were 1, 3, and 10 Hz (results at 1 Hz only are shown), the deformation was 50 μm, and the maximal applied strength was 1 N. Shear storage modulus (G′), shear loss modulus (G′′), and the loss tangent (tan𝛿 = G′′/G′) were calculated using the Mettler Toledo STARTe software during DMTA scans.

### 4.4. Differential Scanning Calorimetry (DSC)

The ice nucleation temperature of *H. rhodopensis* leaves was analyzed with a differential scanning calorimeter (DSC 822e; Mettler Toledo, Greifensee, Switzerland), following a method similar to that described by Fernández-Marín et al. [19,79]. Samples from either leaves at full turgor or at 55% of their RWC were sealed in aluminum pans and characterized under constant liquid nitrogen flow (20 mL min^−1^). Samples were first equilibrated at 0 °C and then cooled at a rate of 0.05 °C min^−1^ down to −15 °C. Subsamples of the same set as that used for DSC scans were used to estimate absolute water content. Before the measurements, the DSC equipment was calibrated with zinc, indium, and pure water as standards. All weights were recorded using a Mettler Toledo 0.1 mg precision balance. DSC measurements were performed on at least seven samples per treatment.

### 4.5. Electrolyte Leakage 

Electrolyte leakage from leaf tissues (0.1:10 ratio of leaf tissue to dH_2_O) was measured with a conductivity meter (EC 215, Hanna Instruments, Woonsocket, RI, USA). After 24 h incubation of leaf disks in double-distilled water on an orbital shaker (OS-20, Boeco & CO GmbH, Hamburg, Germany), the conductivity (μS cm^−1^) of the solution was measured. The maximum leakage of the tissue was determined after boiling the leaves for 15 min at 100 °C. The results are expressed as a percentage of the maximum leakage.

### 4.6. Malondialdehyde Content (MDA)

MDA was determined according to Esterbauer and Cheeseman [80]; 250 mg leaves were homogenized at 4 °C in 2.5 mL of 0.1% trichloracetic acid (TCA) and centrifuged at 15,000× *g* for 30 min at 4 °C. The reaction mixture contained 0.5 mL of the supernatant, 0.5 mL of 0.1% TCA, and 1 mL of 0.5% thiobarbituric acid in 20% (*w*/*v*) TCA. This solution was boiled for 30 min at 95 °C in a water bath, and after centrifugation at 4000× *g* for 10 min, the absorbance was read spectrophotometrically (Multiskan Spectrum, Thermo Scientific, Waltham, MA, USA) at 532 and 600 nm for the determination of MDA. The data were calculated on a dry weight basis.

### 4.7. Proline Content

Proline was determined by the method of Bates et al. [81]; 400 mg leaves were homogenized in 6 mL of 3% aqueous sulphosalicylic acid, and the homogenate was centrifuged at 5000× *g* for 20 min at 4 °C. The extract of 2 mL was mixed with 2 mL of acidified ninhydrin (2.5% ninhydrin dissolved into 3:2 (*v*/*v*) glacial acetic acid:orthophosphoric acid mixture) and 2 mL of glacial acetic acid. The reaction mixtures were kept in a water bath at 100 °C for 1 h, and after cooling in ice, 4 mL toluene was added. The chromophore containing toluene was separated, and the absorbance was read spectrophotometrically at 520 nm (Spekol 11, Carl Zeiss, Jena, Germany). The amount of proline was calculated using a standard curve and expressed on a dry weight basis.

### 4.8. Carbohydrate Analysis

Soluble sugars and organic acids were analyzed in lyophilized leaf samples (ca. 0.5 g) using gas chromatography with flame ionization detection (GC-FID) on a Varian CP 3800) according to Georgieva et al. [30]. Briefly, the lyophilized samples were extracted three times with 4 mL of 1:1 solution of imidazole buffer (0.05 M, pH 7.2) and ethanol. Combined extracts were dried under an air stream and re-suspended in 200 μL of anhydrous pyridine and silanized with 400 μL of derivatization solution (hexamethyldisilazane to trimethylchlorosilane, 3:1) for 1.5 h at 55 °C. Compounds were separated on a capillary column (SGE BPX5 25 m, ID 0.25 mm df, 0.25 μm SGE). In both the natural environmental and controlled conditions, samples were taken at midday.

### 4.9. Fatty Acid Analysis 

The fatty acid quantification method was adapted from Zanetti et al. [82]. Approximately 100 mg of fresh leaf sample was homogenized with 2 mL of chloroform–methanol (1:2; *v*/*v*) solution for 24 h. After adding 1 mL of diethyl ether, the mixture was centrifuged (9000× rpm for 6 min), and the organic phase was collected. The pellet was re-extracted a total of three times. Supernatants were combined, dried completely under a nitrogen stream, and re-dissolved in 400 μL of heptane for methylation, according to Liu [83]. In detail, 200 μL of a 1 M NaOH solution in methanol was added and vortexed for 2 min. After adding 200 μL of 1N H_2_SO_4_ solution in methanol and vortexed for 2 min, the extract was heated at 40 °C for 30 min. The supernatant (1 μL) was injected in a CP-3800 gas chromatograph (Varian, Walnut Creek, CA, USA) equipped with FID and a 30 m long, ID 0.32 mm, df 0.25 μm Mega-10 high polarity column (Mega, Milan, Italy). The injector and detector temperature was 260 °C, and the fatty acids were separated by a gradient of temperatures as follows: initial temperature 140 °C for 1 min, from 140 °C to 200 °C with an increment of 5 °C min^−1^, from 200 °C to 260 °C with an increase of 10 °C m^−1^, final temperature 260 °C for 3 min.

### 4.10. Total Leaf Protein Extraction, SDS-PAGE and Western Blot Analysis 

Total leaf proteins were extracted in two ways: (1) in sample buffer [21] for the Western blot of ELIPs and dehydrins or (2) in potassium–phosphate buffer according to Mladenov et al. [84] with some modifications for the Western blot of sucrose synthase 1 (SUS1) [22]. The protein content of the supernatant was determined according to Bradford [85]. Isolated samples were separated on 12% (SUS1 and dehydrins) or 16% (ELIPs) SDS-PAGE (SE260 Mighty Small II, Hoefer, Holliston, MA, USA) according to Laemmli [86], modified by adding 8.7% glycerol to stacking and separating gels using a constant current of 20 mA per gel. Each lane contains 30 µg total leaf protein. Using semi-dry transfer (TE70X, Hoefer, Holliston, MA, USA) the proteins were blotted on nitrocellulose membrane for 90 min at a current of 1 mA cm^−2^. Prestained protein standard (Precision Plus Protein™ Dual Color Standards, Bio-Rad, Hercules, CA, USA) was used for monitoring electrophoresis separation and transfer efficiency. Blots were probed with primary antibodies against SUS1 (AS15 2830, Agrisera, Vännäs, Sweden), ELIPs (AS06 147A, Agrisera, Vännäs, Sweden), dehydrin K- (AS07 206A, Agrisera, Vännäs, Sweden), Y- and S-segments [87]. Horseradish peroxidase-conjugated goat anti-rabbit secondary antibody was used (AS09 602, Agrisera, Vännäs, Sweden). The resulting bands were visualized by chemiluminescence, and signals were recorded on X-ray Blue films (Carestream Dental LLC, Atlanta, GA, USA). Films were scanned using an Epson Perfection V850 PRO scanner and quantified using Gel-Pro Analyzer software (Media Cybernetic, Rockville, MD, USA).

### 4.11. Statistical Analysis

The experiment under controlled conditions in a growth chamber was repeated twice and samples were taken from six different plant rosettes. During ex situ experiments, leaves were sampled from at least ten different tufts at each time point. Comparison of means was made by the Fisher least significant difference (LSD) test at *p* ≤ 0.05 following ANOVA. A statistical software package (*StatGraphics Plus*, version 5.1 for Windows, The Plains, VA, USA) was used.

The Pearson’s correlation analysis was performed to better explore the linear association between examined physiological and biochemical parameters, using the “rcorr” function from the “Hmisc” R package. This analysis was also conducted using our unpublished data characterizing the photochemical activity of photosystem II (Fv/Fm and ΦPSII).

## 5. Conclusions

**Figure 11:**
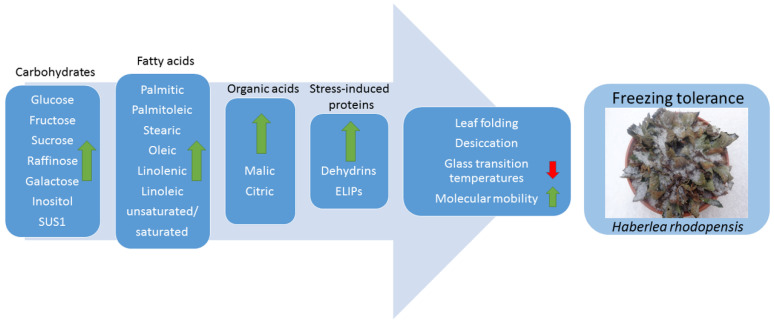
Schematic representation of the protective strategies used by *H. rhodopensis* to achieve freezing tolerance.

Overall, our data shows that changes in membrane lipid composition, accumulation of sugars, and synthesis of stress-induced proteins are significantly activated for the adaptation of *H. rhodopensis* to both cold and freezing temperatures. The contribution of the examined metabolites to freezing tolerance differed not only in the absolute values but also in the progression of their modulation, reflecting an overall reprogramming of the correspondent metabolic pathways. In particular, the analysis of the dynamic and interdependencies of central carbohydrate metabolism allowed us to capture a more comprehensive understanding of the acquisition of freezing tolerance in this resurrection species. In response to chilling temperatures, as typically experienced during autumn, *H. rhodopensis* accumulates preferentially hexoses, mainly glucose and fructose, in its leaves. If this accumulation of hexoses might prepare plants for freezing temperatures, a prominent role of raffinose and sucrose progressively emerge in the survival of subzero freezing conditions during the coldest winter months. This pattern was paralleled by an elevated ratio of unsaturated/saturated fatty acids and significant qualitative and quantitative changes in stress-induced proteins, namely dehydrins and ELIPs. Taking into account the well-documented vitrification properties of both soluble sugars and dehydrins, as well as their interactive functions, their pronounced increase in leaves of acclimated *H. rhodopensis* during freezing-dehydration also indicates that the formation of a glassy state is likely to occur. In Figure 11 are summarized the protective strategies used by *H. rhodopensis* to achieve freezing tolerance. In addition, the observed changes of both sugars and stress-induced proteins in response to low temperatures suggest that the ability of *H. rhodopensis* to cold acclimate and tolerate freezing temperatures relies on some resurrection-linked traits. Our results further suggest that future research on the biochemical adaptation of *H. rhodopensis* to low temperatures should better investigate the relative dynamics of the metabolite modulation and their interconnection. Their specific functions, depending on the subcellular localization, also represent a crucial point in dissecting their specific impact on the enhancement of protective mechanisms, such as photoprotection.

## Figures and Tables

**Table 1:** Fatty acid composition measured in leaves of *Haberlea rhodopensis* collected after exposure to low temperatures during cold acclimation (CA; period: 7–28 November), after short-term exposure to freezing temperatures (FS; 30 November), after longer-term exposure to freezing temperatures when significant desiccation occurred (FS + D; period: 5–14 December) and after recovery of plants in early spring (R; period: 22 March). Data represent the mean of *n* = 4. The same letters within a graph indicate no significant differences assessed by Fisher’s LSD test (*p* ≤ 0.05) after performing ANOVA. Percentages of RWC of leaves is reported at each time point.

	Control	CA		FS	FS + D			R
Fatty acid	5 May	7 November	28 November	30 November	5 December	8 December	14 December	22 March
	RWC 86%	RWC 80%	RWC 82%	RWC 65%	RWC 35%	RWC 20%	RWC 12%	RWC 86%
Palmitic (16:0)	40.36 ± 1.54 d	38.84 ± 0.65 cd	36.45 ± 1.02 bc	36.77 ± 2.73 bc	36.73 ± 0.11 bc	36.38 ± 0.90 bc	27.61 ± 2.74 a	35.47 ± 0.60 b
Palmitoleic (16:1)	0.34 ± 0.01 d	0.12 ± 0.02 ab	0.15 ± 0.01 b	0.34 ± 0.08 d	0.06 ± 0.01 a	0.16 ± 0.01 b	0.24 ± 0.04 c	0.59 ± 0.04 e
Stearic (18:0)	28.81 ± 1.63 b	29.88 ± 1.14 b	32.30 ± 1.70 b	23.82 ± 1.02 a	24.42 ± 1.25 a	24.53 ± 0.49 a	22.93 ± 3.23 a	24.67 ± 2.84 a
Oleic (18:1)	12.87 ± 1.56 d	9.61 ± 0.31 bc	9.61 ± 0.63 bc	16.57 ± 1.84 e	5.97 ± 0.80 a	6.06 ± 0.15 a	7.58 ± 1.32 ab	10.32 ± 1.52 c
Linoleic (18:2)	12.51 ± 1.43 a	14.22 ± 0.88 ab	12.50 ± 1.14 a	12.60 ± 1.91 a	16.61 ± 2.13 bc	18.23 ± 0.30 c	23.20 ± 4.23 d	17.21 ± 1.33 bc
Linolenic (18:3)	4.20 ± 0.48 a	6.74 ± 0.75 ab	8.01 ± 1.61 b	9.17 ± 1.62 bc	15.68 ± 2.24 de	14.28 ± 0.78 d	17.57 ± 2.43 e	11.09 ± 1.96 c
Arachidic (20:0)	0.53 ± 0.07 cd	0.51 ± 0.03 c	0.45 ± 0.02 bc	0.66 ± 0.07 e	0.44 ± 0.01 bc	0.13 ± 0.02 a	0.64 ± 0.10 de	0.36 ± 0.05 b
Behenic (22:0)	0.28 ± 0.05 b	0.09 ± 0.01 a	0.53 ± 0.08 c	0.07 ± 0.02 a	0.08 ± 0.01 a	0.23 ± 0.01 b	0.23 ± 0.05 b	0.31 ± 0.10 b

**Table 2:** Relative changes in concentration of different carbohydrates and organic acids measured in leaves of *Haberlea rhodopensis* collected after exposure to low temperatures during cold acclimation (CA; period: 7–28 November), after short-term exposure to freezing temperatures (FS; −10 °C, 30 November), after longer-term exposure to freezing temperatures when significant desiccation occurred (FS + D; period: 5–14 December) and after recovery of plants in early spring (R; period: 22 March). Data are expressed in arbitrary units (fold changes) normalized to the amount found in control samples collected in May. Percentages of RWC of leaves are reported at each time point.

		Control	CA		FS*	FS + D			R
Group	Compounds	5 May	7 November	28 November	30 November	5 December	8 December	14 December	22 March
		RWC 86%	RWC 80%	RWC 82%	RWC 65%	RWC 35%	RWC 20%	RWC 12%	RWC 86%
Carbohydrates	Raffinose	1.00	1.16	1.44	2.81	1.84	6.84	16.53	0.71
	Sucrose	1.00	0.85	1.54	1.17	2.93	4.36	15.87	1.13
	Glucose	1.00	2.58	3.91	4.22	6.09	8.56	5.07	2.79
	Fructose	1.00	1.76	3.41	3.06	4.42	8.00	5.16	2.23
	Galactose	1.00	2.12	2.31	4.07	3.31	3.31	2.14	2.74
	Inositol	1.00	2.44	2.87	1.72	2.58	2.89	2.83	2.81
Organic acids	Malic acid	1.00	1.41	1.48	0.61	2.43	3.12	3.51	1.03
	Citric acid	1.00	1.38	1.37	0.42	1.29	1.28	3.15	1.10

## Data Availability

All data are contained within the article.

## Supplementary Materials

The following supporting information can be downloaded at: https://www.mdpi.com/article/10.3390/ijms232315050/s1, Figure S1: Changes in malondialdehyde (MDA) content during cold acclimation of *H. rhodopensis*, after exposure to −10 °C for 12 h and 24 h recovery at day/night temperature of 20/15 °C under temperature-controlled conditions; Figure S2: Changes in proline content during cold acclimation of *H. rhodopensis*, after exposure to −10 °C for 12 h and 24 h recovery at day/night temperature of 20/15 °C under temperature-controlled conditions; Figure S3: Changes in the content of fructose, glucose, sucrose and raffinose after exposure to low temperatures during cold acclimation, freezing stress, freezing-induced desiccation and after recovery of *H. rhodopensis* in early spring under natural ex situ environmental conditions; Figure S4: Changes in the content of galactose, inositol, malic and citric acids after exposure of *H. rhodopensis* to low temperatures during cold acclimation, freezing stress, freezing-induced desiccation and after recovery of plants in early spring under natural ex situ environmental conditions; Figure S5: Changes in the content of fructose, glucose, sucrose and raffinose after cold acclimation of *H. rhodopensis*, exposure to −10 °C for 12 h and 24 h recovery at day/night temperature of 20/15 °C under temperature-controlled conditions; Figure S6: Changes in mean concentration of carbohydrates and organic acids measured in leaves of *H. rhodopensis* from the Control under temperature-controlled conditions; Figure S7: Representative Western blots of dehydrins (top) after cold acclimation of *H. rhodopensis*, exposure to −10 °C for 12 h and 24 h recovery at day/night temperature of 20/15 °C under temperature-controlled conditions by Western blot using antibodies against the conserved K-, S- and Y-segment of the proteins. The Ponceau S staining (bottom) of the membranes after blotting are presented below the Western blots; Figure S8: Representative Western blots of ELIPs (left) after cold acclimation of *H. rhodopensis*, exposure to −10 °C for 12 h and 24 h recovery at day/night temperature of 20/15 °C. The Ponceau S staining (right) of the membrane after blotting is presented on the right; Table S1: Fatty acid composition after cold acclimation of *H. rhodopensis*, exposure to −10 °C for 12 h and 24 h recovery at day/night temperature of 20/15 °C under temperature-controlled conditions; Table S2: Sampling points, daily maximum and minimum temperature data for experiments in natural (ex situ) and under controlled conditions.
