# Supplementary material for: Protective Strategies of Haberlea rhodopensis for Acquisition of Freezing Tolerance: Interaction between Dehydration and Low Temperature
Source: Int J Mol Sci. 2022 Nov 30;23(23):15050. doi: 10.3390/ijms232315050 (PMC9739172; doi:10.3390/ijms232315050)
Supplement: Supplementary file 1 [file ijms-23-15050-s001.zip › ijms-1971212-supplementary.pdf]

Supplementary material

Protective strategies of *Haberlea rhodopensis* for acquisition of freezing tolerance: interaction between dehydration and low temperature

Katya Georgieva [1,*], Gergana Mihailova [1], Beatriz Fernández-Marín [2], Gianpaolo Bertazza [3], Annalisa Govoni [3], Miren Irati Arzac [4], José Manuel Laza [5], José Luis Vilas [5], José Ignacio García-Plazaola [4], Francesca Rapparini [3]

[1] Institute of Plant Physiology and Genetics, Bulgarian Academy of Sciences, Acad. G. Bonchev Str., Bl. 21, 1113 Sofia, Bulgaria
[2] Department of Botany, Ecology and Plant Physiology, University of La Laguna (ULL), 38200 Tenerife, Spain
[3] Bioeconomy Institute (IBE), Department of Bio-Agrifood Science (DiSBA), National Research Council (CNR), Via P. Gobetti 101, I-40129 Bologna, Italy
[4] Department of Plant Biology and Ecology, University of the Basque Country (UPV/EHU), Barrio Sarriena s/n, 48940 Bilbao, Spain
[5] Department of Physical Chemistry, University of the Basque Country (UPV/EHU), Barrio Sarriena s/n, 48940 Bilbao, Spain
* Correspondence: georgieva.katya.m@gmail.com; Tel.: +359-2-979-2620

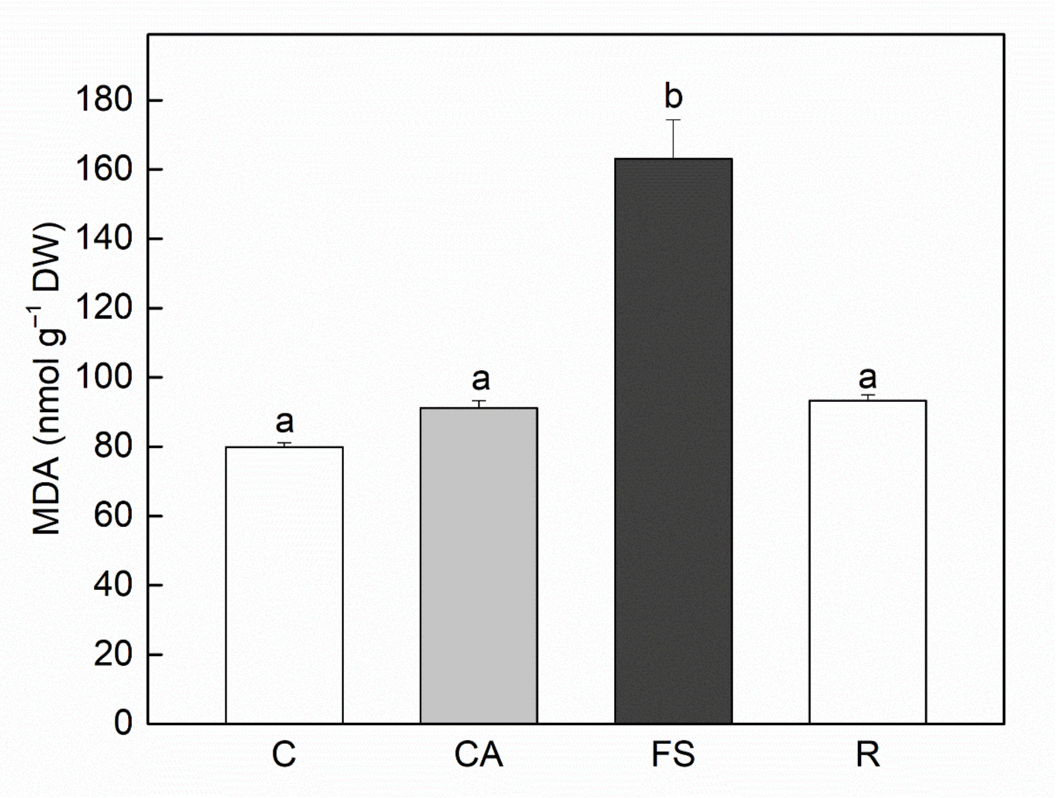

Figure S1. Changes in malondialdehyde (MDA) content during cold acclimation of *Haberlea rhodopensis* (CA, 2 weeks at 5 °C), after exposure to –10 °C for 12 h (FS) and 24 h recovery at day/night temperature of 20/15 °C (R) under temperature-controlled conditions. Data represent the mean of $n = 12$. The same letters within a graph indicate no significant differences assessed by Fisher's LSD test ($p \leq 0.05$) after performing ANOVA.

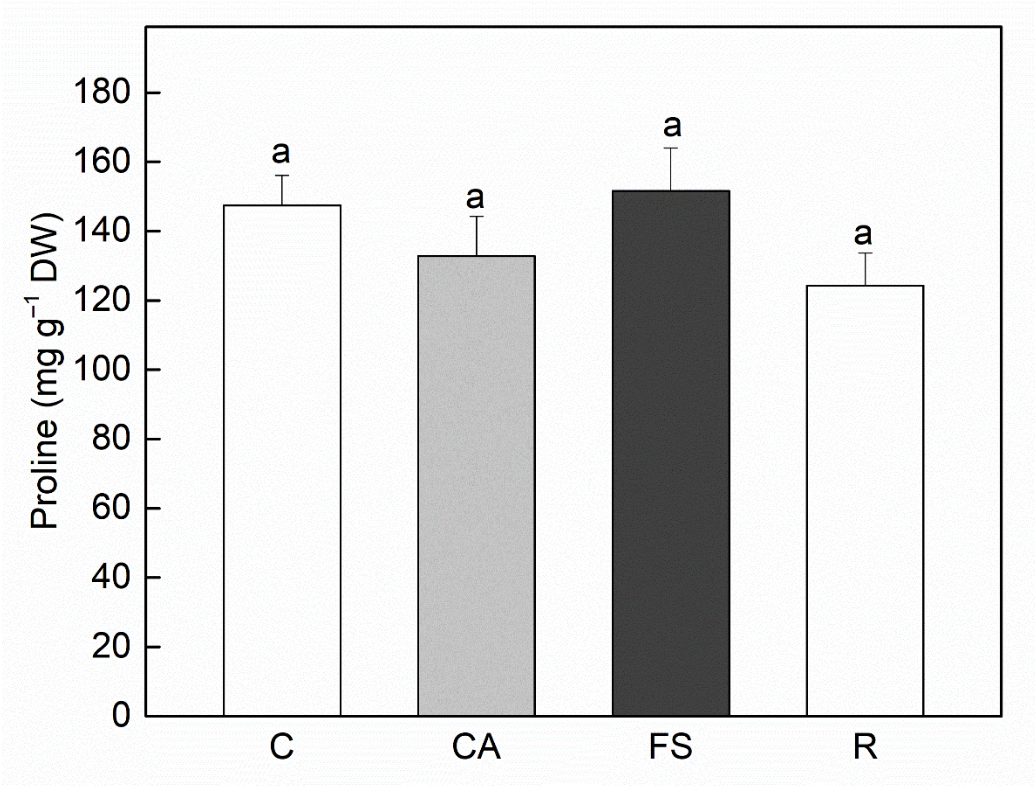

Figure S2. Changes in proline content during cold acclimation of *Haberlea rhodopensis* (CA, 2 weeks at 5 °C), after exposure to –10 °C for 12 h (FS) and 24 h recovery at day/night temperature of 20/15 °C (R) under temperature-controlled conditions. Data represent the mean of $n = 12$. The same letters within a graph indicate no significant differences assessed by Fisher's LSD test ($p \leq 0.05$) after performing ANOVA.

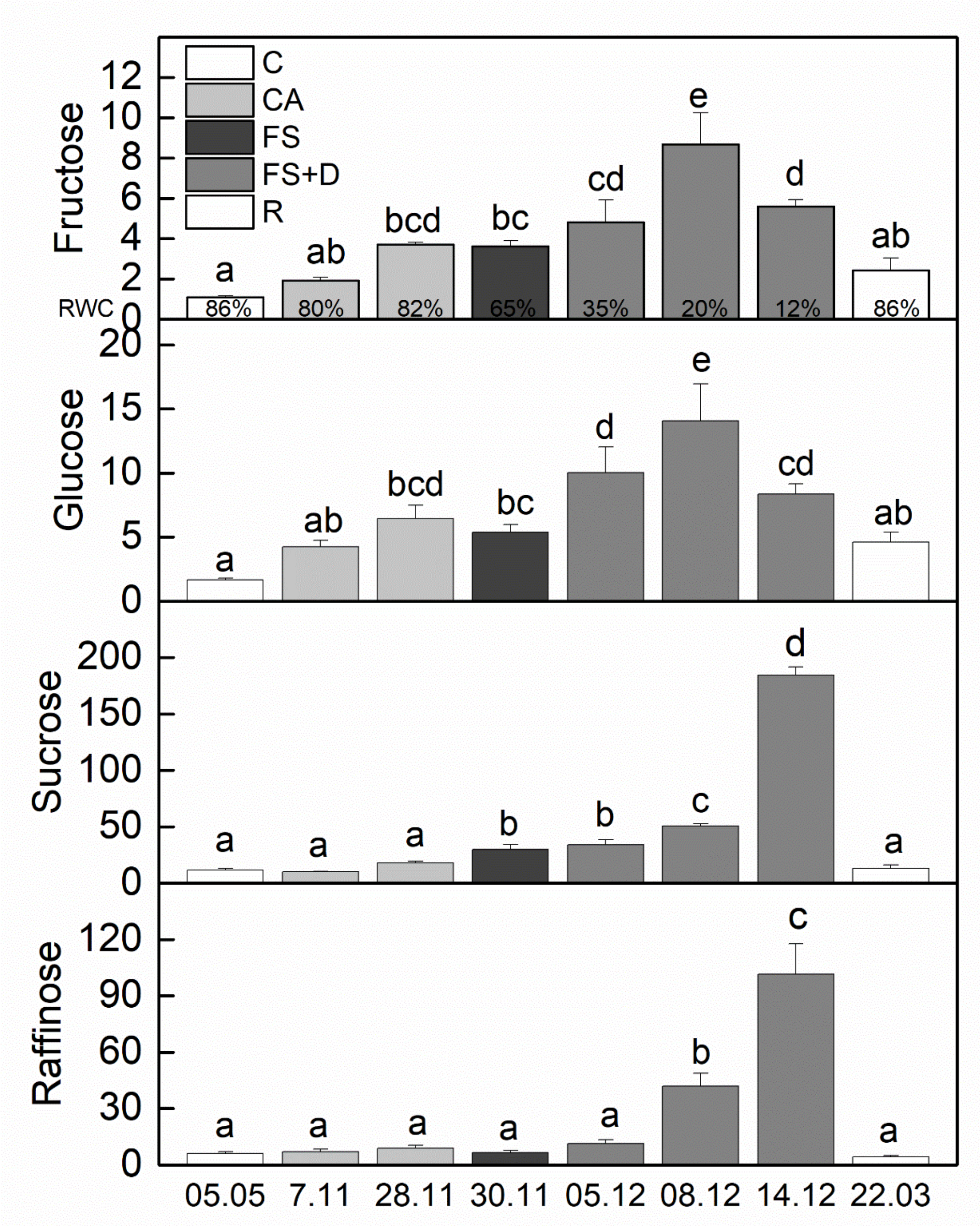

Figure S3. Changes in the content of fructose, glucose, sucrose and raffinose (mg g^{-1} DW) after exposure to low temperatures during cold acclimation (CA, period: 07–28 November), freezing stress (FS, –10 °C, 30 November), freezing-induced desiccation (FS+D, period: 05–14 December) and after recovery of *H. rhodopensis* in early spring (R; period: 22 March) under natural ex situ environmental conditions. Percentages at the bottom of the columns show the RWC of plants at each sampling point. Data represent the mean of $n = 4$. The same letters within a graph indicate no significant differences assessed by Fisher's LSD test ($p \leq 0.05$) after performing ANOVA.

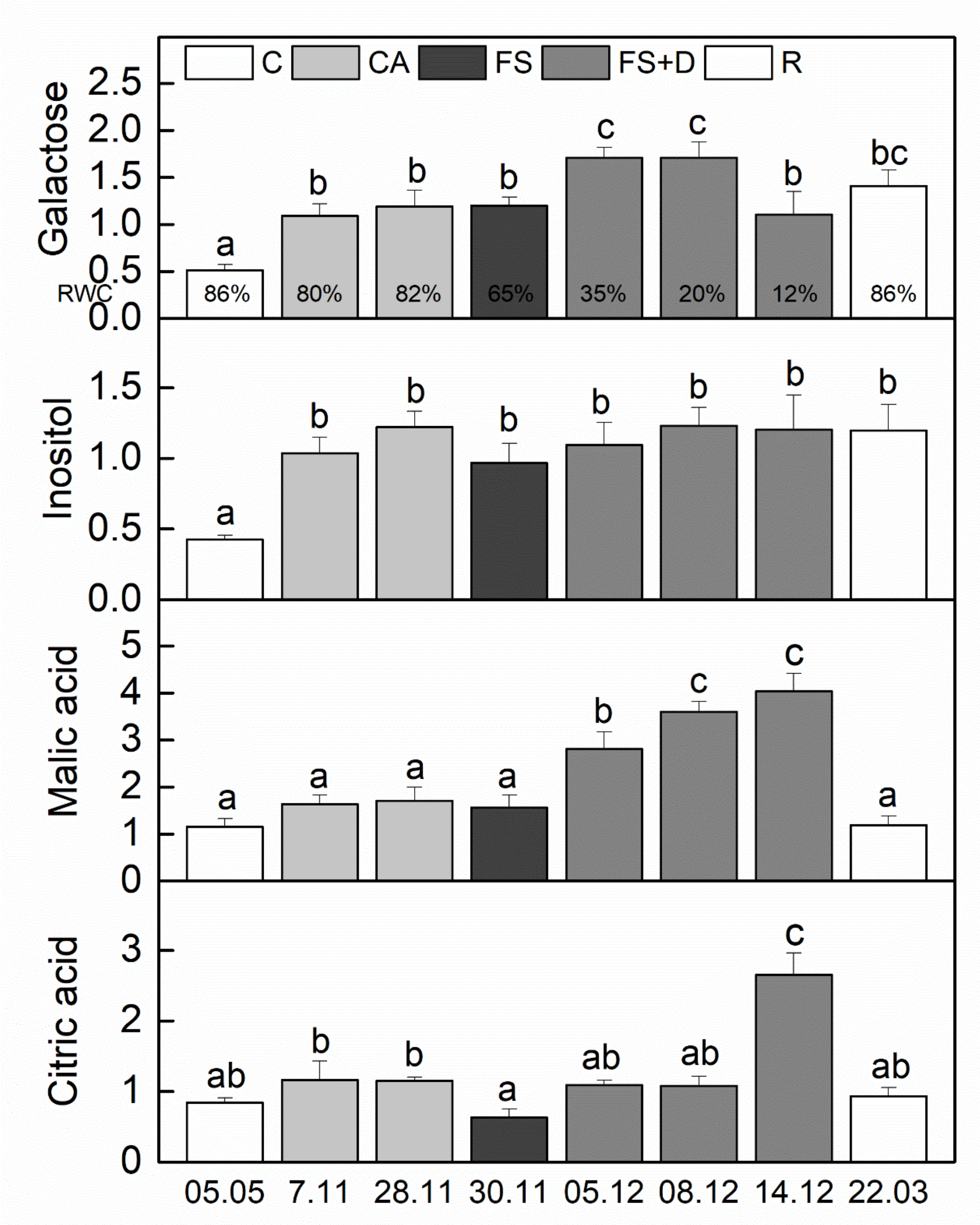

Figure S4. Changes in the content of galactose, inositol, malic and citric acids (mg g^{-1} DW) after exposure of *Haberlea rhodopensis* to low temperatures during cold acclimation (CA, period: 07–28 November), freezing stress (FS, –10 °C, 30 November), freezing-induced desiccation (FS+D, period: 05–14 December) and after recovery of plants in early spring (R; period: 22 March) under natural ex situ environmental conditions. Percentages at the bottom of the columns show the RWC of plants at each sampling point. Data represent the mean of $n = 4$. The same letters within a graph indicate no significant differences assessed by Fisher's LSD test ($p \leq 0.05$) after performing ANOVA.

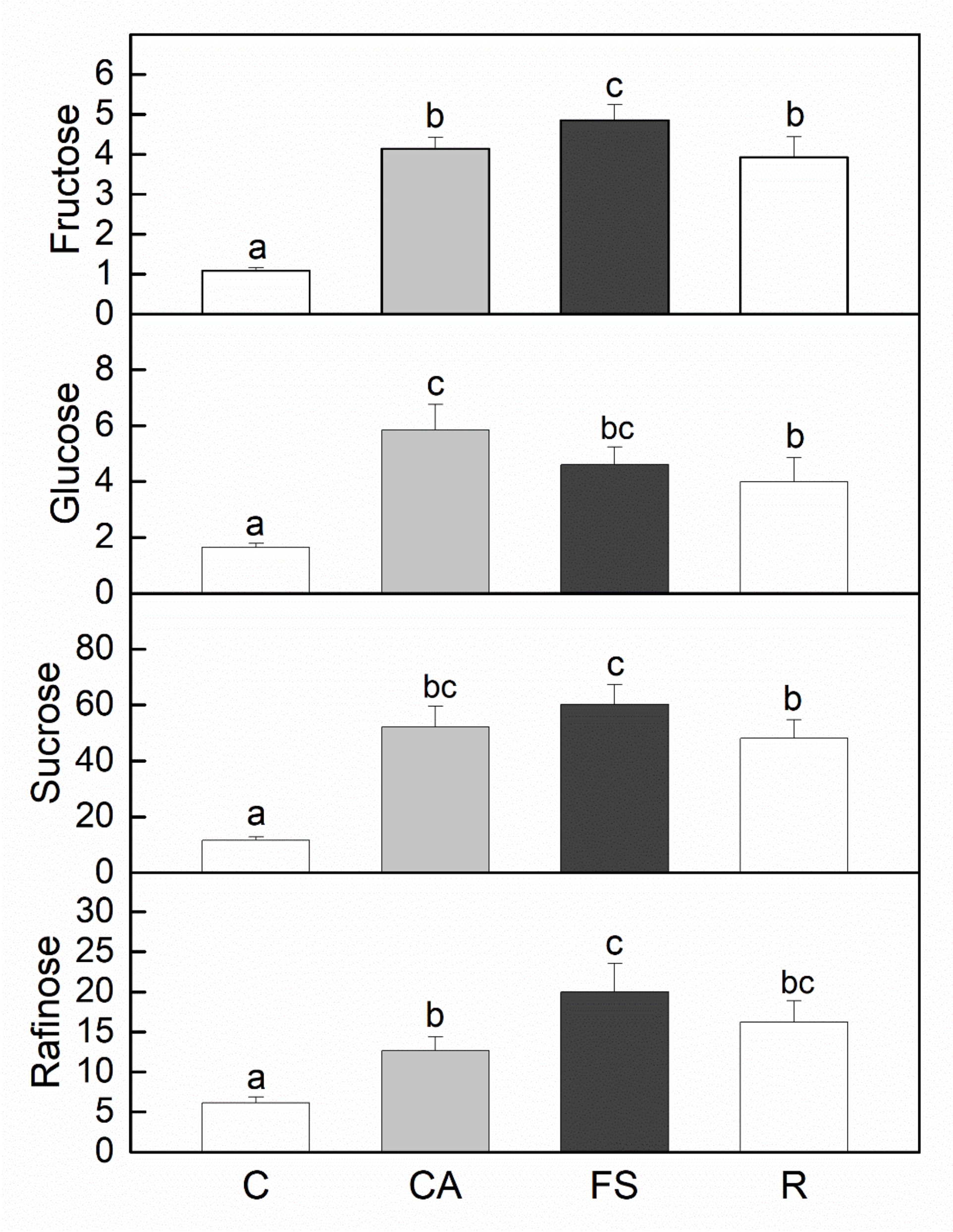

Figure S5. Changes in the content of fructose, glucose, sucrose and raffinose (mg g^{-1} DW) after cold acclimation of *Haberlea rhodopensis* (CA, 2 weeks at +5 °C), exposure to –10 °C for 12 h (FS) and 24 h recovery at day/night temperature of 20/15 °C (R) under temperature-controlled conditions. Data represent the mean of $n = 12$. The same letters within a graph indicate no significant differences assessed by Fisher's LSD test ($p \leq 0.05$) after performing ANOVA.

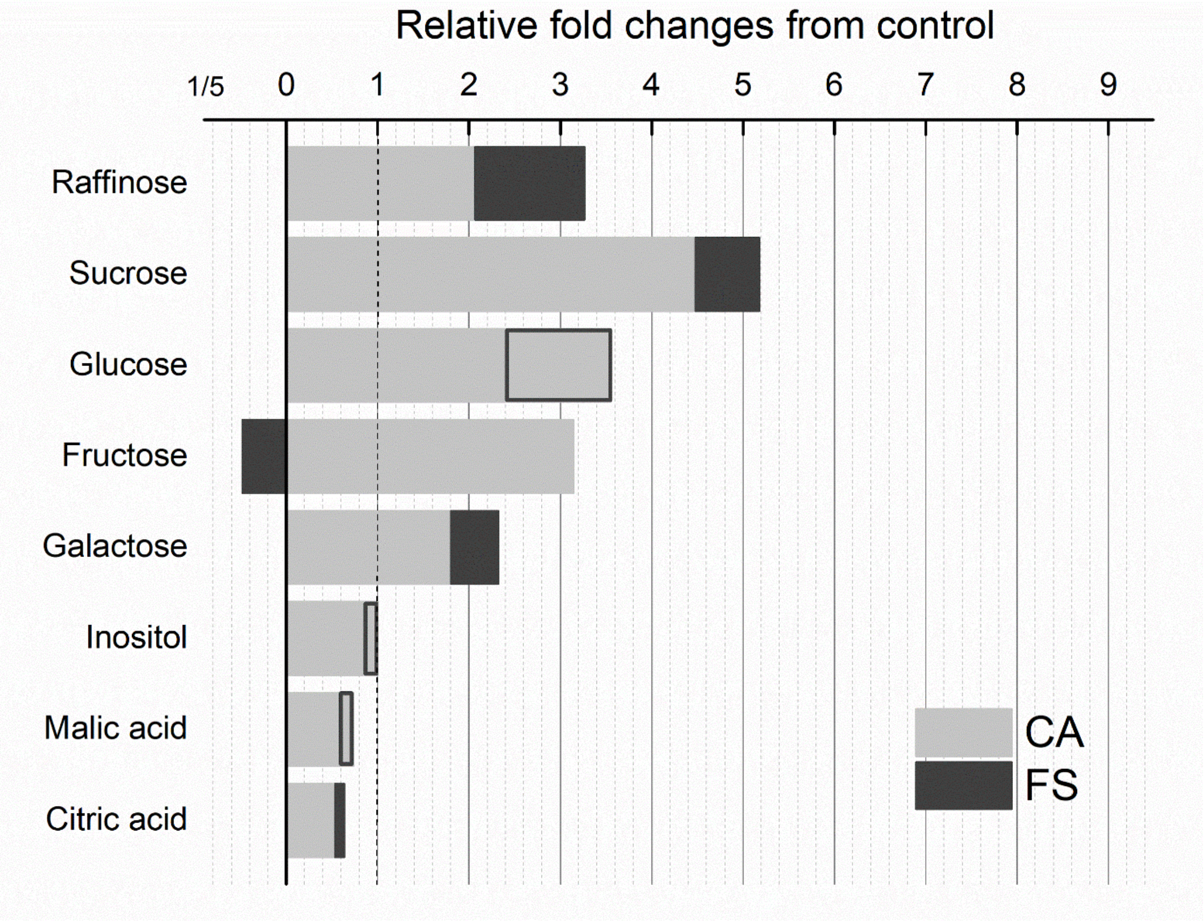

Figure S6. Changes in mean concentration of carbohydrates and organic acids measured in leaves of *Haberlea rhodopensis* from the Control under temperature-controlled conditions. Light grey bars indicate changes between Control and cold acclimation (CA; at +5 °C for 2 weeks). Dark grey bars indicate changes from CA to short-term exposure to freezing temperatures (FS; exposure to –10 °C for 12 h). The bars for compounds for which the trend between one phase was opposed to that observed during the precedent transition have only the grey outlines.

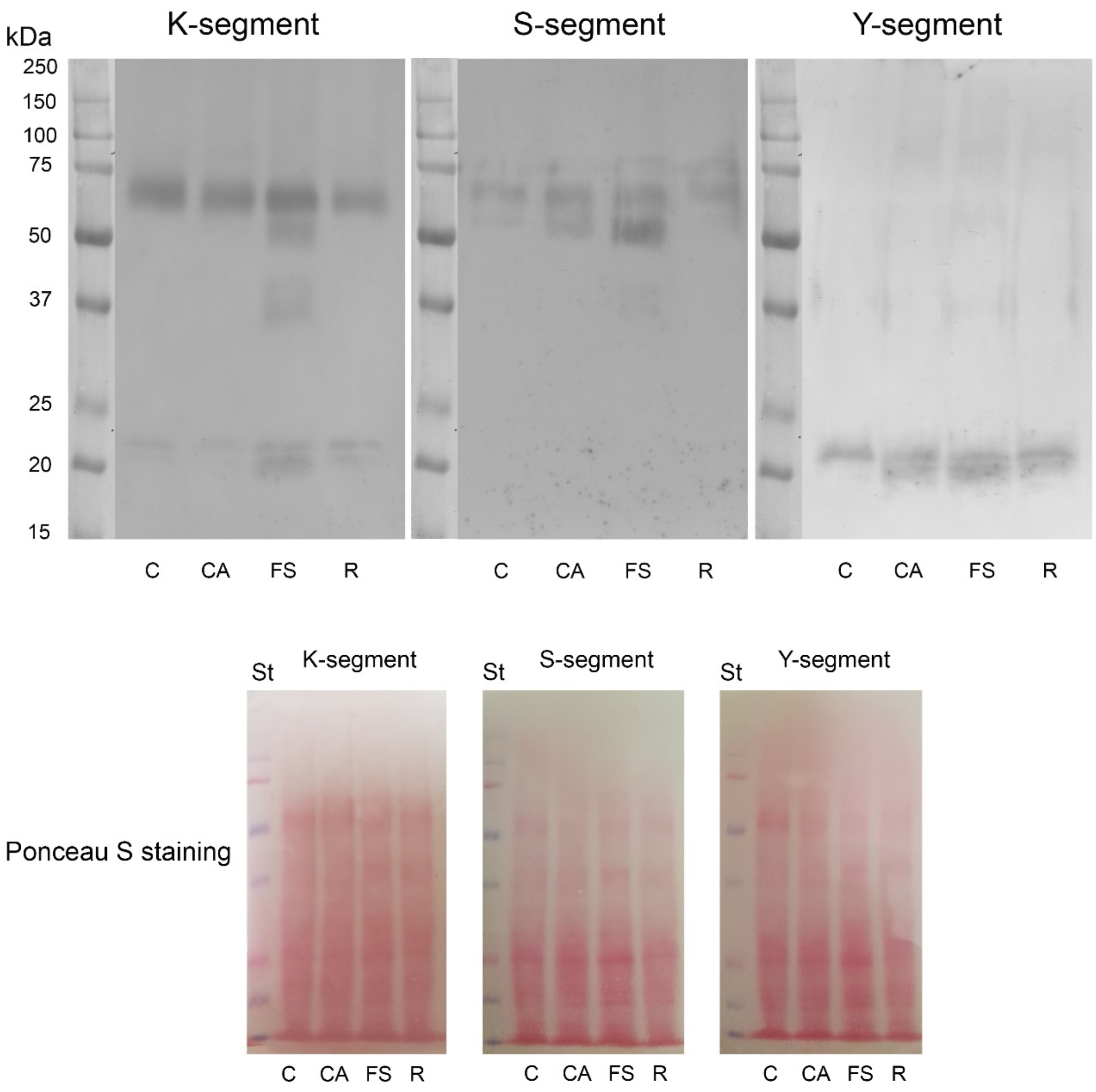

Figure S7. Representative Western blots of dehydrins (top) after cold acclimation of *Haberlea rhodopensis* (CA, 2 weeks at 5 °C), exposure to –10 °C for 12 h (FS) and 24 h recovery at day/night temperature of 20/15 °C (R) under temperature-controlled conditions by Western blot using antibodies against the conserved K-, S- and Y-segment of the proteins. 30 μg protein was applied per lane. The Ponceau S staining (bottom) of the membranes after blotting are presented below the Western blots. St: Precision Plus Dual Color Protein™ Prestained Standards (Bio-Rad, Hercules, CA, USA).

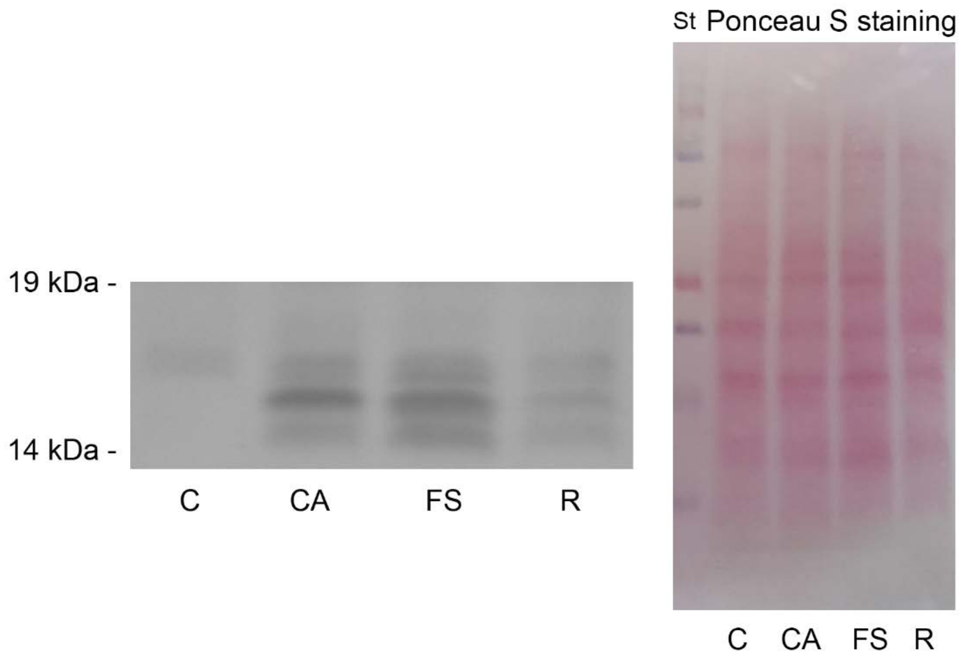

Figure S8. Representative Western blots of ELIPs (left) after cold acclimation of *Haberlea rhodopensis* (CA, 2 weeks at 5 °C), exposure to –10 °C for 12 h (FS) and 24 h recovery at day/night temperature of 20/15 °C (R)**.** 30 µg protein was applied per lane. The Ponceau S staining (right) of the membrane after blotting is presented on the right. St: Precision Plus Dual Color Protein™ Prestained Standards (Bio-Rad, Hercules, CA, USA).

Table S1. Fatty acid composition after cold acclimation of *Haberlea rhodopensis* (CA, 2 weeks at 5 °C), exposure to –10 °C for 12 h (FS) and 24 h recovery at day/night temperature of 20/15 °C (R) under temperature-controlled conditions. Data represent the mean of $n = 6$. The same letters within a graph indicate no significant differences assessed by Fisher's LSD test ($p \leq 0.05$) after performing ANOVA.

**Fatty acid**	**Control**	**CA**	**FS**	**R**
Palmitic (16:0)	40.36 ± 1.54b	37.48 ± 2.90ab	35.25 ± 1.04a	38.47 ± 2.41ab
Palmitoleic (16:1)	0.34 ± 0.01ab	0.26 ± 0.08a	0.51 ± 0.06c	0.42 ± 0.03bc
Stearic (18:0)	28.81 ± 1.63ab	34.80 ± 2.70c	23.99 ± 2.37a	29.80 ± 2.78bc
Oleic (18:1)	12.87 ± 1.56b	6.05 ± 0.86a	12.58 ± 2.48b	12.12 ± 1.75b
Linoleic (18:2)	12.51 ± 1.43a	10.75 ± 1.36a	17.39 ± 1.22b	11.74 ± 2.24a
Linolenic (18:3)	4.20 ± 0.48a	9.72 ± 1.64b	9.25 ± 0.83b	6.48 ± 1.69a
Arachidic	0.53 ± 0.07a	0.64 ± 0.23ab	0.95 ± 0.04b	0.71 ± 0.26ab
Behenic (22:0)	0.28 ± 0.05b	0.30 ± 0.10b	0.09 ± 0.01a	0.26 ± 0.06b

Table S2. Sampling points, daily maximum and minimum temperature data for experiments in natural (ex situ) and under controlled conditions.

ex situ natural conditions											
date	5.5.2016	7.11.2016	17.11.2016	28.11.2016	30.11.2016	1.12.2016	5.12.2016	8.12.2016	14.12.2016	30.1.2017	22.3.2017
Variant	C	CA	CA	CA*	FS**	FS+D	FS+D	FS+D	FS+D	FS+D	R
RWC (%)	86	80	80	82	65	55	35	20	12	10	86
Min Temp (°C)	12	13	2	-2	-9	-1	-5	-3	-4	-11	6
Max Temp (°C)	23	23	6	9	-2	3	6	4	3	-2	22
Mean Temp (°C)	17.5	18	4	3.5	-5.5	1	0.5	0.5	-0.5	-6.5	14

Controlled climatic chamber				
Variant	C	CA*	FS**	R
RWC (%)	91	90	86	89
Min Temp (°C)	15	5	-9	15
Max Temp (°C)	20	5	-11	20
Mean Temp (°C)	17.5	5	-10	17.5
	2 weeks	2 weeks	12 h	24 h

C	Control
CA	Cold acclimated
FS	Freeezing stress
FS+D	Freeezing stress + dehydration
R	Recovery

(dark green)	>10°C
(light green)	6-10°C
(light blue)	0-5°C
(blue)	-5/0°C
(dark blue)	-10/-5°C

(red box) Similar conditions between natural and controlled experiments
* after CA
** short-term exposure to temperature of - 10°C
